# Economical bisphenol-based dibenzocyclobutene resins with excellent thermal and dielectric properties for high-frequency communication applications

**DOI:** 10.1039/d6ra06919c

**Published:** 2026-09-07

**Authors:** Honghui Cheng, Lei Wu, Jiafeng Di, Hua Lai, Yanyu Li, Chi Zhang, Xiao Wu, JinRong Ye

**Affiliations:** a College of Chemistry and Materials Science, Hengyang Normal University Hengyang China laixhua163@163.com; b Tongyu Advanced Materials (Guangdong) Co., Ltd Zhaoqing China yanyu_li@tyadmt.com

## Abstract

Benzocyclobutene (BCB) resins have recently gained significant attention as dielectric materials, yet most suffer from high costs. Herein, five economical bisphenol-based dibenzocyclobutene resins (BCB-A, BCB-AF, BCB-F, BCB-B and BCB-O) were readily synthesized from 4-bromobenzocyclobutene and commercial bisphenols *via* an Ullmann reaction. Upon thermal curing, the corresponding crosslinked polymers were obtained. All cured BCB resins exhibited high glass transition temperature (*T*_g_) values of 287–329 °C, excellent thermal stability with 5% weight loss temperatures (*T*_5%_) of 408–445 °C, and CTE values as low as 23.8 ppm °C^−1^. The cured resins also exhibited good flexural properties, with flexural strengths of 63.7–98.4 MPa and flexural moduli of 2.2–2.7 GPa. Notably, they also demonstrated favorable high-frequency dielectric properties at 10 GHz, with dielectric constant (*D*_k_) values of 2.54–2.83 and dissipation factor (*D*_f_) values of 2.32 × 10^−3^ to 3.63 × 10^−3^. Considering the low cost of bisphenols, the facile Ullmann reaction and the good overall performance, these bisphenol-based dibenzocyclobutene resins are promising candidates for high-frequency communication applications.

## Abstract 2

Economical bisphenol-based dibenzocyclobutene resins with excellent thermal stability and dielectric properties for high-frequency communication applications.

## Graphical abstract

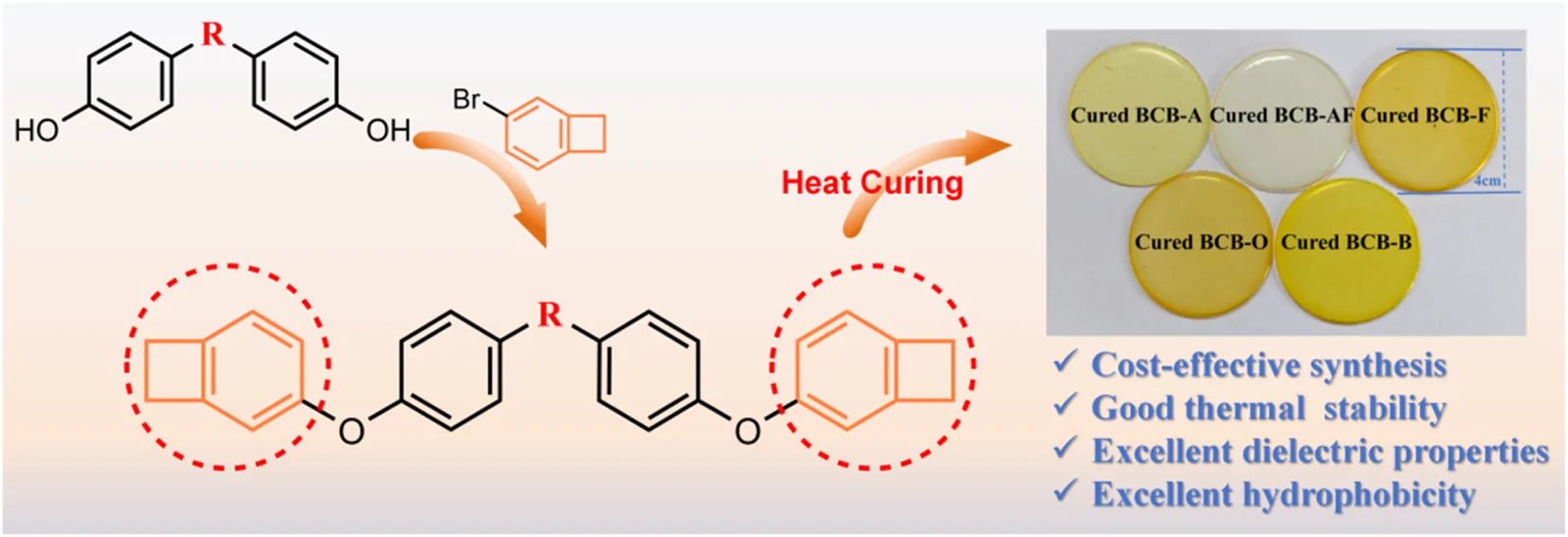

## 1 Introduction

Electronic devices used in 5G communication, AI servers, robotics and electric vehicles are evolving toward higher operating frequencies, faster data transmission and greater integration densities.^1,2^ These trends impose unprecedented demands on printed circuit board (PCB) materials, particularly the need for lower *D*_k_ and *D*_f_.^3^ Traditional epoxy resins, whose molecular structures contain numerous polar groups after curing, exhibit high *D*_k_ (3.2–4.5) and high *D*_f_ (>0.02). Although modification can reduce the *D*_k_ of conventional epoxy resins to 2.39–3.77, some modified epoxy systems still exhibit *D*_f_ values of 0.018 or higher at high frequencies.^4–6^ Such dielectric losses can still cause appreciable attenuation during high-frequency signal transmission. Hydrocarbon resins and polytetrafluoroethylene (PTFE) or polyphenylene oxide (PPO) are regarded as promising low-k candidates for high-frequency applications, with representative systems exhibiting *D*_k_ values of approximately 2.1–2.7 and *D*_f_ values of 3.5 × 10^−4^ to 6.3 × 10^−3^ in the GHz range.^7^ More recently, a high-silica-fiberglass-cloth/PTFE composite exhibited a *D*_k_ of 2.41 and a *D*_f_ of 4.9 × 10^−3^ at 10 GHz, while PPO/benzoxazine thermosets showed *D*_k_ values of 2.29–2.47 and *D*_f_ values of 5.50–6.83 × 10^−3^ at the same frequency.^8,9^ Although these studies demonstrate the favorable dielectric properties of such materials, further suppression of dielectric loss remains desirable to minimize high-frequency signal attenuation. Moreover, their practical applications may be restricted by insufficient thermal stability and/or poor processability.^10,11^ Therefore, developing readily processable thermosetting resins that combine low *D*_k_ and low *D*_f_ with high thermal stability remains highly desirable. Benzocyclobutene (BCB) resins have attracted considerable attention as dielectric materials because of their favorable processability, high thermal stability, and excellent dielectric properties.^12–14^ Consequently, BCB resins have become an important class of materials for high-frequency dielectric applications.^15,16^

The synthesis of BCB resins has been accomplished *via* various methods, including hydrosilylation,^17,18^ Suzuki coupling,^19–22^ Heck reaction,^15,23^ Grubbs reaction,^24^ Ullmann reaction^15,22,24–26^ and Grignard reaction.^25,27^ However, most of these methods suffer from high costs associated with the use of precious metal catalysts and silicon-containing or fluorine-containing raw materials, which constrain their commercial applicability. In contrast, the Ullmann reaction—specifically, the C–O coupling of 4-bromobenzocyclobutene with inexpensive, commercially available bisphenols—offers great promise for practical applications. For example, Zhao *et al.* synthesized a dibenzocyclobutene resin (AB) from an adamantyl-containing bisphenol and 4-bromobenzocyclobutene *via* a modified Ullmann–Ma reaction, using *N*_1_,*N*_2_-bis(thiophen-2-ylmethyl)oxalamide as the ligand and copper(i) iodide (CuI) as the catalyst.^28^ Wu *et al.* prepared another dibenzocyclobutene resin from 2,6-dimethyl phenol-DCPD novolac (DCPDNO) and 4-bromobenzocyclobutene through Ullmann coupling with copper(i) chloride (CuCl) and 2,2,6,6-tetramethyl-3,5-heptanedione as the ligand.^29^ Nevertheless, the use of expensive cesium carbonate (Cs_2_CO_3_) and an oxalamide ligand considerably undermines the practical prospects of this method.

To achieve economical production of BCB resins, the following strategies were adopted in this study. First, five commercially available and low-cost bisphenols were employed as starting materials: 2,2-bis(4-hydroxyphenyl)propane (BPA), 4,4′-(hexafluoroisopropylidene)diphenol (BPAF), bis(4-hydroxyphenyl)methane (BPF), 4,4′-dihydroxydiphenyl ether (BPO), and 4,4′-dihydroxybiphenyl (BPB). Second, based on a previously reported Ullmann coupling procedure,^22^ CuI was employed as the catalyst, 1-butylimidazole as an inexpensive ligand and potassium carbonate (K_2_CO_3_) as the base, with 4-bromobenzocyclobutene serving as both the reactant and the solvent. Consequently, the five dibenzocyclobutene resins were readily obtained in moderate yields as crystalline solids. These resins exhibited suitable melting points and thermal crosslinkability, providing a favorable processing window for subsequent thermal curing. Owing to their relatively low-polarity structures, the corresponding thermally crosslinked polymers are expected to possess good dielectric properties. Additionally, the influence of the different bisphenol linking groups on the structure–property relationship was examined by comparative analysis. Herein, we report the details of this study.

## 2 Experimental section

### 2.1 Materials

4-Bromobenzocyclobutene was synthesized *via* bromination of BCB, which was prepared by flash vacuum pyrolysis (FVP). This synthetic procedure has been successfully scaled up to the 10 kg scale in our laboratory. BPA, BPF, BPAF, BPO and BPB were provided by Shanghai Bide Pharmaceutical Co., Ltd. 1-Butylimidazole and CuI were purchased from Beijing InnoChem Science & Technology Co., Ltd. and anhydrous K_2_CO_3_ was purchased from Sinopharm Chemical Reagent Co., Ltd.

### 2.2 Measurements

^1^H NMR spectra were recorded on an AVANCE III 400 spectrometer using CDCl_3_ as the solvent and tetramethylsilane (TMS) as the internal standard. Fourier transform infrared (FTIR) spectra were obtained on an IR Prestige-21 instrument (Shimadzu, Japan) with KBr pellets in the range of 600–4000 cm^−1^. Differential scanning calorimetry (DSC) was performed on a TA Instruments Q20 under a nitrogen atmosphere at a heating rate of 10 °C min^−1^ from 40 to 350 °C to determine the melting points of the resins. Thermogravimetric analysis (TGA) was conducted on a Mettler Toledo TGA 2 under a nitrogen atmosphere from 40 to 800 °C at a heating rate of 10 °C min^−1^. Dynamic mechanical analysis (DMA) was carried out on a TA Instruments DMA 850 under a nitrogen atmosphere to measure the glass transition temperature (*T*_g_) and storage modulus. The heating rate was 3 °C min^−1^ over a temperature range of 100–350 °C. Samples of cured resins for DMA testing had dimensions of 60 × 15 × 0.8 mm. Thermomechanical analysis (TMA) was performed on a Mettler Toledo TMA/SDTA 2^+^ to determine the coefficient of thermal expansion. The measurement was conducted from 45–400 °C at a heating rate of 5 °C min^−1^. Sample dimensions were 5 × 5 × 0.8 mm. The flexural properties of the cured samples were evaluated by a three-point bending test using a CMT6103 electronic universal testing machine. Rectangular specimens with dimensions of 80 × 10 × 4 mm were tested at a crosshead speed of 10 mm min^−1^ with a support span of 64 mm. Dielectric properties (*D*_k_ and *D*_f_) of cured resins were measured at 5 and 10 GHz using a Keysight E5063A network analyzer under ambient conditions (23 ± 2 °C, 45 ± 5% relative humidity). Samples for dielectric measurements were prepared as circular disks with a diameter of 40 mm and a thickness of 0.6 mm. Water uptake was determined by immersing the cured specimens in boiling water for 24, 48, and 72 h. After immersion, the specimens were removed, gently wiped to remove surface water, and immediately weighed. For each formulation, water uptake and dielectric properties were measured using three independently prepared specimens.

### 2.3 Synthesis of dibenzocyclobutene resins

#### 2.3.1 BCB-AF

4-Bromobenzocyclobutene (73.22 g, 0.40 mol), BPAF (33.63 g, 0.10 mol), CuI (3.81 g, 0.02 mol), anhydrous K_2_CO_3_ (55.28 g, 0.40 mol), and 1-butylimidazole (12.42 g, 0.10 mol) were added to a dry 500 mL round-bottom three-necked flask that had been purged with argon. The mixture was heated to 140 °C and stirred for 16 h. After cooling to room temperature, the mixture was filtered. The organic phase was washed with water and dried over anhydrous sodium sulfate. The filtrate was then concentrated under reduced pressure. The crude product was purified by column chromatography followed by recrystallization to afford a powdery crystalline solid (36.65 g, 67.8% yield). The excess 4-bromobenzocyclobutene could be recovered and reused. ^1^H NMR (400 MHz, CDCl_3_, *δ*): 7.31 (d, *J* = 9.0 Hz, 4H), 7.04 (d, *J* = 8.1 Hz, 2H), 6.95–6.88 (m, 6H), 6.81 (d, *J* = 2.1 Hz, 2H), 3.16 (s, 8H). ^13^C NMR (126 MHz, CDCl_3_, *δ*): 159.02, 154.95, 146.97, 141.56, 131.58, 126.91, 124.04, 119.39, 116.75, 115.35, 29.09, 28.99. ^19^F NMR(471 MHz, CDCl_3_, *δ*): −64.06 (s, 6F).

#### 2.3.2 BCB-A

The synthesis followed the same procedure as described for BCB-AF, using BPA as the bisphenol. The product was obtained in a yield of 65.6%. ^1^H NMR (400 MHz, CDCl_3_, *δ*): 7.17 (d, *J* = 8.9 Hz, 4H), 7.00 (d, *J* = 7.9 Hz, 2H), 6.88 (d, *J* = 8.9 Hz, 6H), 6.76 (s, 2H), 3.14 (s, 8H), 1.67 (s, 6H). ^13^C NMR (126 MHz, CDCl_3_, *δ*): 156.42, 155.98, 146.68, 144.99, 140.40, 127.91, 123.77, 118.53, 117.58, 114.43, 42.02, 31.07, 29.05, 28.92.

#### 2.3.3 BCB-F

The synthesis followed the same procedure as described for BCB-AF, using BPF as the bisphenol. The product was obtained in a yield of 66.1%. ^1^H NMR (400 MHz, CDCl_3_, *δ*): 7.11 (d, *J* = 8.5 Hz, 4H), 6.98 (d, *J* = 7.9 Hz, 2H), 6.87 (dd, *J* = 19.6, 8.9 Hz, 6H), 6.73 (s, 2H), 3.90 (s, 2H), 3.12 (s, 8H). ^13^C NMR (126 MHz, CDCl_3_, *δ*): 156.55, 156.47, 146.71, 140.38, 135.58, 129.97, 123.79, 118.42, 118.34, 114.32, 40.41, 29.05, 28.92.

#### 2.3.4 BCB-O

The synthesis followed the same procedure as described for BCB-AF, using BPO as the bisphenol. The product was obtained in a yield of 55.8%. ^1^H NMR (400 MHz, CDCl_3_, *δ*): 7.01 (d, *J* = 8.1 Hz, 2H), 6.96 (s, 8H), 6.87 (dd, *J* = 7.9, 2.3 Hz, 2H), 6.75 (d, *J* = 2.3 Hz, 2H), 3.14 (s, 8H). ^13^C NMR (126 MHz, CDCl_3_, *δ*): 156.92, 153.49, 152.92, 146.77, 140.29, 123.83, 119.75, 118.01, 113.91, 29.06, 28.92.

#### 2.3.5 BCB-B

The synthesis followed the same procedure as described for BCB-AF, using BPB as the bisphenol. The product was obtained in a yield of 53.1%. ^1^H NMR (400 MHz, CDCl_3_, *δ*): 7.49 (d, *J* = 8.7 Hz, 4H), 7.04 (dd, *J* = 8.3, 4.3 Hz, 6H), 6.92 (d, *J* = 7.9 Hz, 2H), 6.81 (s, 2H), 3.17 (s, 8H). ^13^C NMR (126 MHz, CDCl_3_, *δ*): 157.58, 156.22, 146.83, 140.72, 135.18, 128.04, 123.90, 118.70, 118.39, 114.63, 29.09, 28.97.

### 2.4 Curing process of dibenzocyclobutene resins

The bisphenol-based dibenzocyclobutene resins were placed in a quartz mould and heated to melting under 1 atm in a nitrogen atmosphere. They were then cured following a programmed heating profile: 180 °C for 0.5 h, followed by 220 °C for 3 h, and finally 240 °C for 2 h. After cooling to room temperature, intact resin blocks were obtained. Details of the sample dimensions for each measurement are provided in Section 2.2 (Measurements).

## 3 Results and discussion

### 3.1 Synthesis and characterization

**Scheme 1:**
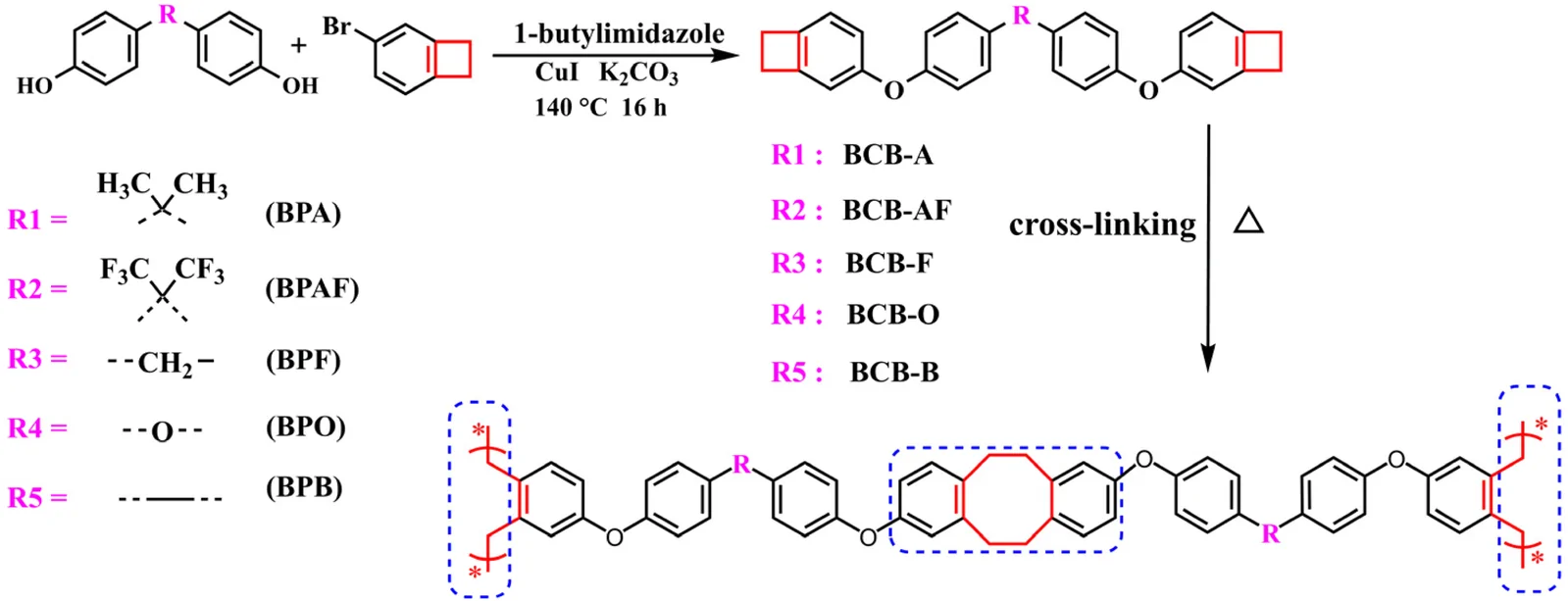
Procedure for synthesis and thermal cross-linking of dibenzocyclobutene resins.

Bisphenol-based dibenzocyclobutene resins were synthesized from 4-bromobenzocyclobutene and inexpensive bisphenols *via* a copper-catalyzed Ullmann coupling reaction. During the optimization of the catalytic conditions, the Ullmann–Ma reaction with oxalamide ligands^30^ was found to selectively afford the mono-BCB products (Fig. S1). Accordingly, an imidazole/CuI-catalyzed method^31^ was adopted, as illustrated in Scheme 1. 4-Bromobenzocyclobutene served as both the reactant and the solvent, owing to its high boiling point (up to 220 °C). When the reaction temperature was increased to 140 °C, the yield of the dibenzocyclobutene resins exceeded 50%, and the purity was improved *via* column chromatography followed by recrystallization. Notably, in contrast to the Ullmann–Ma reaction, which relies on expensive Cs_2_CO_3_ and oxalamide ligands, the present strategy employed cost-effective 1-butylimidazole and K_2_CO_3_. This offered a significant advantage in reducing the overall cost of bisphenol-based dibenzocyclobutene resins. A detailed raw-material cost analysis of the five BCB resins, together with a comparison with representative previously reported BCB systems, is provided in Table S1 in the SI. The results quantitatively demonstrate the cost advantages associated with the use of commercially available bisphenols, K_2_CO_3_, and 1-butylimidazole in the present synthetic route.

The five bisphenol-based dibenzocyclobutene resins were characterized by ^1^H NMR, ^13^C NMR, and ^19^F NMR. Detailed data are provided in the Experimental Section and in Fig. S2–S12. In the ^1^H NMR spectra, the CH_2_ group on the four-membered ring of the benzocyclobutene unit showed a clear signal at 3.16 ppm. The aromatic protons of the BCB ring appeared at around 6.88, 6.92, and 7.04 ppm. For BCB-AF, the ^19^F NMR spectrum exhibited a characteristic signal at about −64.06 ppm, which is attributed to the CF_3_ group. The ^13^C NMR spectra were fully consistent with the target structures. These results collectively confirm that the five Ullmann-coupling products possess the desired structures and high purity.

### 3.2 Thermal crosslinking reaction

**Fig. 1:**
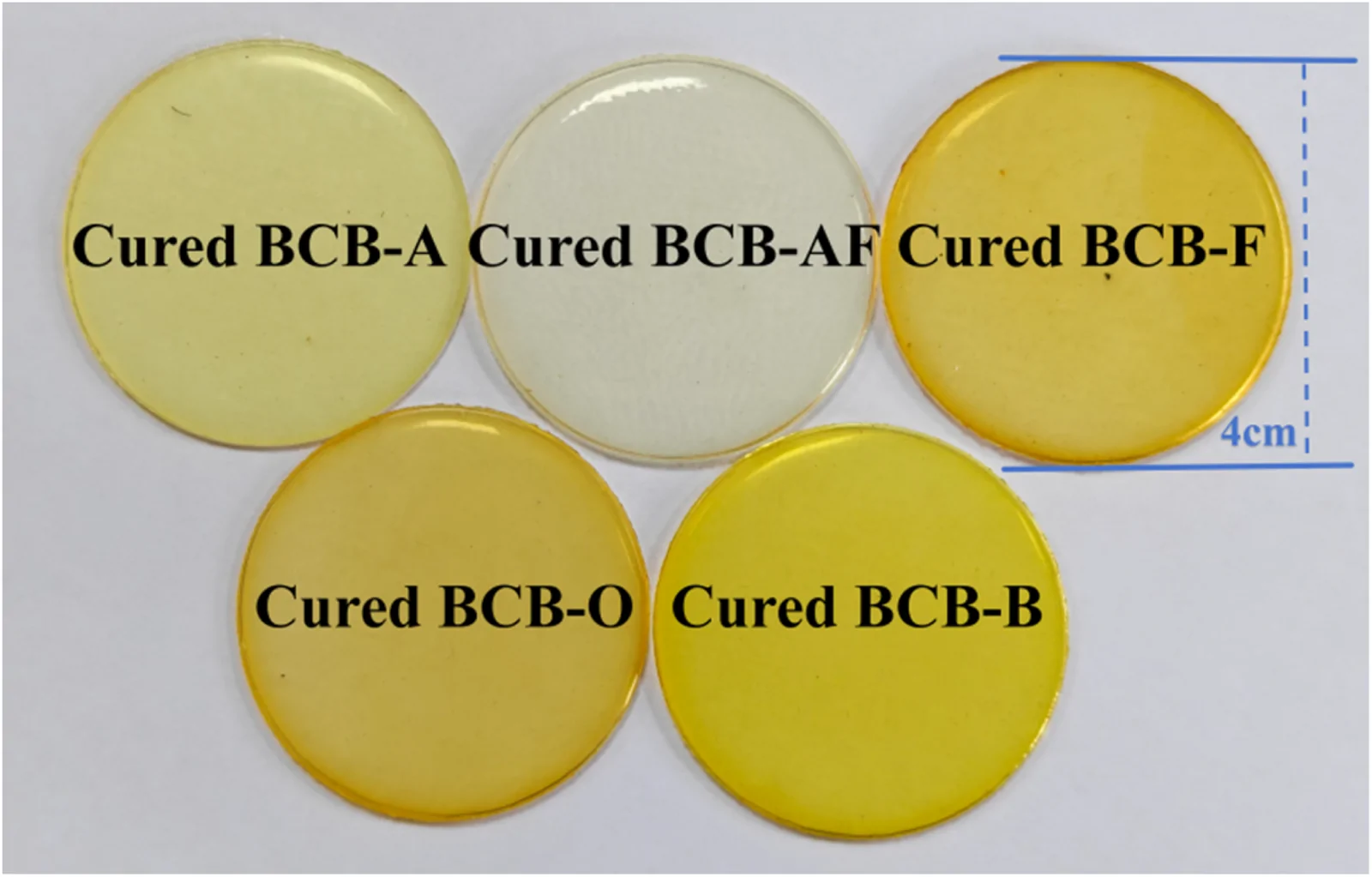
Picture of cured dibenzocyclobutene resins.

The benzocyclobutene (BCB) group contains a strained four-membered ring. This endows it with a useful feature: it is stable at low temperatures but undergoes thermal ring-opening at elevated temperatures. Upon heating to a sufficiently high temperature, the four-membered ring generates an *o*-quinodimethane intermediate, which subsequently undergoes either [4 + 4] cycloaddition to form an eight-membered ring or self-polymerization. All five bisphenol-based dibenzocyclobutene resins (BCB-A, BCB-AF, BCB-F, BCB-O and BCB-B) contain two reactive BCB groups, enabling the formation of a crosslinked network at high temperatures (Scheme 1). This thermal crosslinking behavior enables the formation of highly crosslinked thermosetting networks. The corresponding cured polymers are denoted as Cured BCB-A, Cured BCB-AF, Cured BCB-F, Cured BCB-O and Cured BCB-B, respectively. Photographs of the cured samples are shown in Fig. 1.

**Fig. 2:**
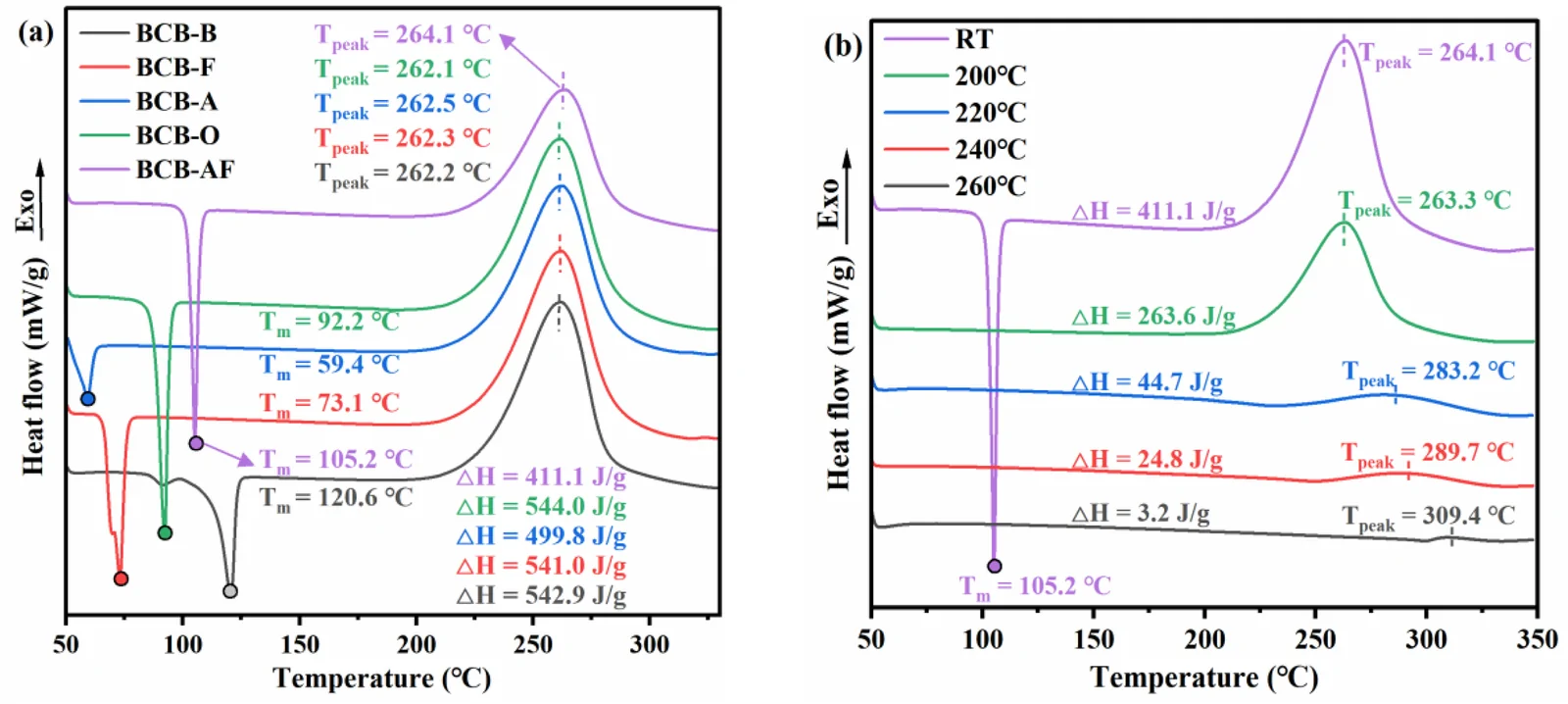
DSC traces of (a) five dibenzocyclobutene resins; (b) samples of BCB-AF precured at different temperatures for 1 h.

**Fig. 3:**
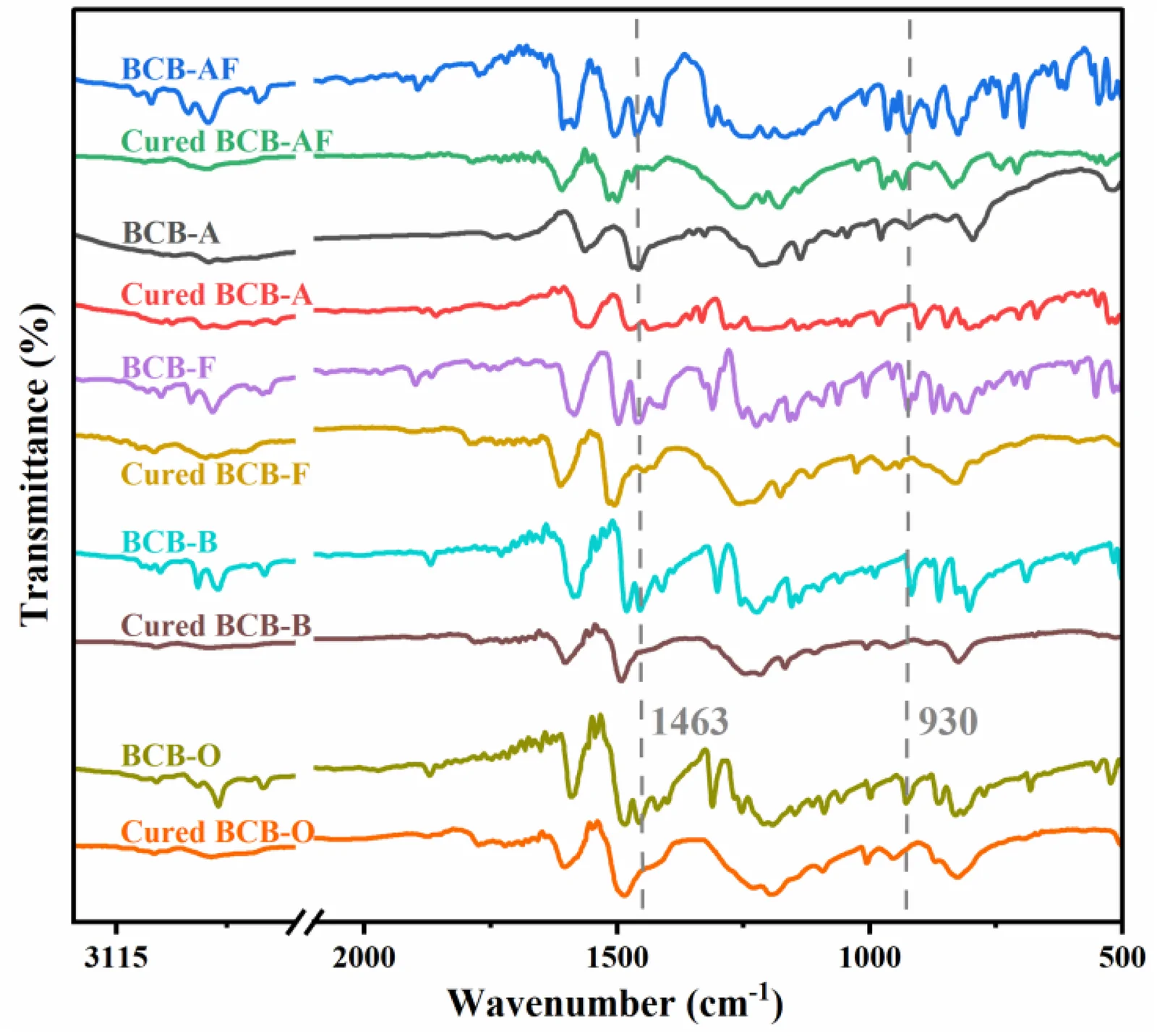
FT-IR spectra of resins and cured resins.

The curing behavior of the five dibenzocyclobutene resins was monitored by DSC, and the results are presented in Fig. 2a. The melting points (*T*_m_) were approximately 120 °C for BCB-B, 73 °C for BCB-F, 59 °C for BCB-A, 92 °C for BCB-O, and 105 °C for BCB-AF. All five resins exhibited similar curing onset temperatures (*T*_onset_) of around 227 °C and peak curing temperatures (*T*_peak_) of around 260 °C. The gap between the melting point and curing onset temperature for each resin exceeds 100 °C, providing a wide processing window that is advantageous for fabrication. Detailed DSC data are summarized in Table 1. In addition, the mass change before and after curing at 1 atm under a nitrogen atmosphere was very small for all five resins, indicating the absence of sublimation or decomposition-driven volatilisation during curing (Table S2). This behavior is highly advantageous for the fabrication of PCBs from these small-molecule thermosetting resins. The curing reactions were further investigated by FTIR spectroscopy. As shown in Fig. 3, the band at 1463 cm^−1^ is predominantly assigned to the in-plane C–H deformation of the methylene groups in the strained four-membered BCB ring, whereas the band at 930 cm^−1^ is attributed to a characteristic skeletal vibration of the four-membered ring, involving predominantly ring C–C stretching coupled with methylene motion.^32–34^ After curing, both bands were markedly attenuated, indicating extensive consumption of the BCB groups through thermally induced ring opening and subsequent crosslinking.

**Table 1:** DSC data for dibenzocyclobutene resins

Resin	*T*_m_ (°C)	*T*_onset_ (°C)	*T*_peak_ (°C)	Δ*H* (J g^−1^)
BCB-A	59.4	227.8	262.5	499.8
BCB-AF	105.2	228.6	264.1	411.1
BCB-F	73.1	227.7	262.3	541.0
BCB-B	120.6	227.2	262.2	542.9
BCB-O	92.2	227.3	262.1	544.0

To determine the appropriate curing temperature, BCB-AF samples were precured at different temperatures for 1 h and then analyzed by DSC (Fig. 2b). As the precuring temperature increased, the curing enthalpy of the samples decreased significantly. Notably, the enthalpy difference between the samples cured at 240 °C and 260 °C was only 21.6 J g^−1^. Furthermore, the FTIR spectra of the samples precured at 240 °C and 260 °C were nearly identical (Fig. S13). These results indicate that BCB-AF was largely cured after 1 h at 240 °C. Accordingly, the following curing protocol was adopted: 180 °C for 0.5 h, 220 °C for 3 h and 240 °C for 2 h.

### 3.3 Thermal properties of cured dibenzocyclobutene resins

**Fig. 4:**
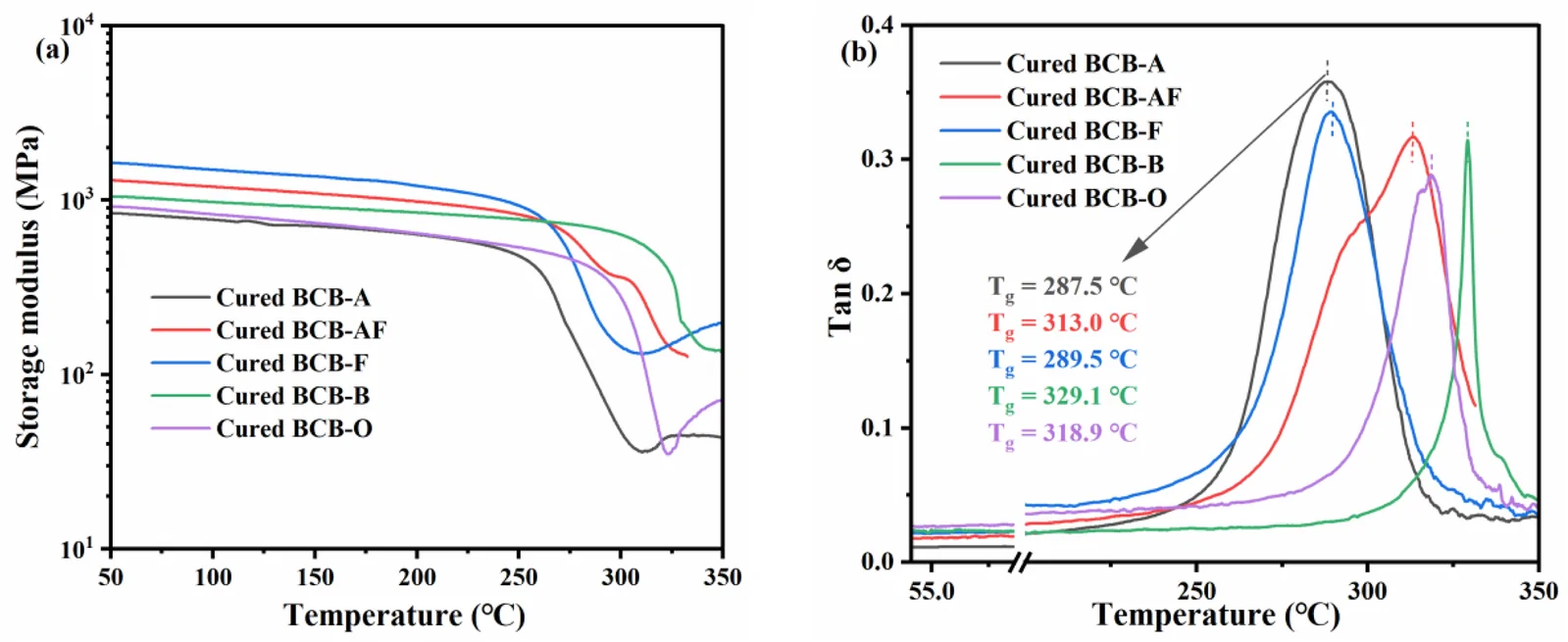
(a) *E*′ and (b) tan *δ versus* temperature curves of cured BCB resins.

The thermal properties of the cured dibenzocyclobutene resins are critical to their dielectric performance in PCB applications, as they ensure stability under elevated temperatures. In this study, the thermal stability of these resins was comprehensively evaluated by DMA, TGA and TMA. The thermomechanical properties of the cured polymers were first analyzed by DMA, and the results are presented in Fig. 4 and summarized in Table 2. At 50 °C, the storage moduli (*E*′) of Cured BCB-A, Cured BCB-AF, Cured BCB-F, Cured BCB-B and Cured BCB-O were 0.85, 1.20, 1.40, 0.98 and 0.89 GPa, respectively. The glass transition temperatures (*T*_g_) of the five samples, determined from the peak of tan *δ*, were 287, 313, 289, 329 and 318 °C, respectively. Previous studies on **M3**, **M4**, and bio-based M1 (ref. 22 and 26) have reported that benzocyclobutene resins bearing three crosslinkable groups can achieve *T*_g_ values as high as 400 °C, owing to the formation of a densely crosslinked network. In contrast, the present dibenzocyclobutene resins contain only two crosslinkable groups per monomer, and therefore exhibit lower *T*_g_ values of around 300 °C. Nevertheless, these values are still substantially higher than those of conventional epoxy resins, which typically have *T*_g_ values below 200 °C.

**Table 2:** Thermal performance of cured resins

Resin	*E*′ at 50 °C (GPa)	*T*_g_ a (°C)	*T*_g_ b (°C)	*T*_peak_ (°C)	*T*_5%_ (°C)	Residual weight (%)	*V*_e_ c (10^3^ mol m^−3^)
Cured BCB-A	0.85	265	287.5	499	408	30%	2.74
Cured BCB-AF	1.20	275	313.0	501	434	34%	8.66*
Cured BCB-F	1.40	268	289.5	503	435	35%	8.93
Cured BCB-B	0.98	283	329.1	501	428	33%	8.96*
Cured BCB-O	0.89	319	318.9	487	445	33%	4.07

a *T*_g_ was determined by TMA.

b *T*_g_ was determined by DMA.

c The crosslink density (*V*_e_) was calculated as *V*_e_ = *E*′/(3*RT*) using *E*′ at *T*_g_ + 30 °C, except for the value marked *, for which the available *E*′ at *T*_g_ + 20 °C was used.

**Fig. 5:**
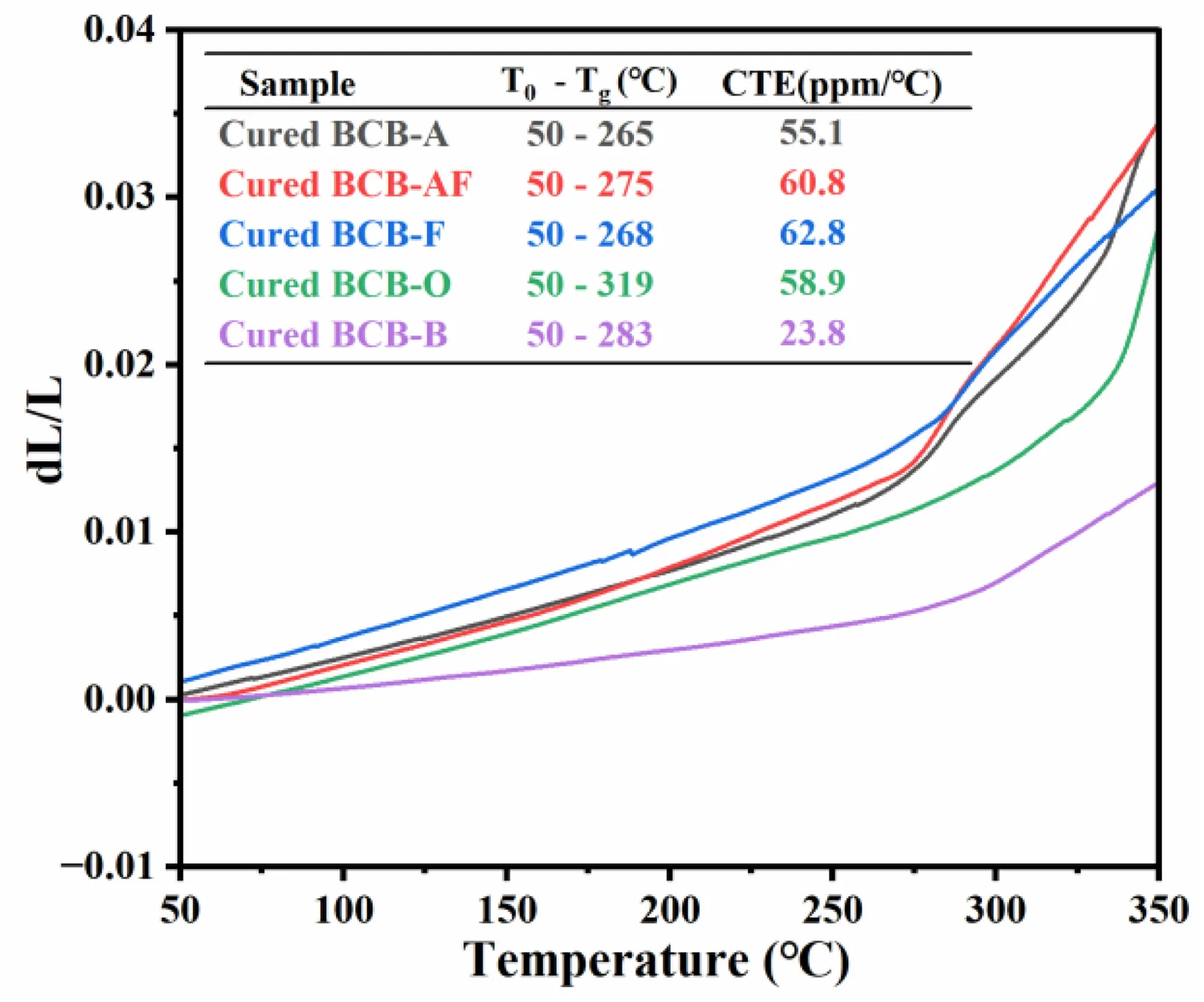
TMA curves of the cured BCB resins at a heating rate of 5 °C min^−1^ in N_2_.

Thermal dimensional stability is a key parameter for evaluating the reliability of polymers at elevated temperatures and can be quantified by the coefficient of thermal expansion (CTE). The thermal dimensional stability of the five cured samples was examined by TMA. The linear CTE values of the cured resins were determined from the slopes of the TMA curves over the temperature range of 50 °C to *T*_g_, as indicated in Fig. 5. For Cured BCB-A, Cured BCB-AF, Cured BCB-F, Cured BCB-O, and Cured BCB-B, the CTEs over the temperature range of 50 to *T*_g_ were 55.1 ppm °C^−1^, 60.8 ppm °C^−1^, 62.8 ppm °C^−1^, 58.9 ppm °C^−1^, and 23.8 ppm °C^−1^, respectively. Among these, Cured BCB-B exhibited the lowest CTE, which is attributable to the rigid biphenyl group. The CTE values of all five cured resins were lower than that of the commercial silicon-containing BCB resin DVS-BCB (65.0 ppm °C^−1^).^35^ They were comparable to those of M3 (37.5 ppm °C^−1^) and M4 (52.7 ppm °C^−1^) containing three BCB groups,^22^ and were superior to those of long-alkyl-chain-containing benzocyclobutene resins (78–208 ppm °C^−1^).^36^

Although all five resins contain two reactive BCB groups and were cured under identical conditions, variations in their bisphenol-derived bridging units led to clear differences in thermomechanical behavior. Previous studies have shown that greater network rigidity and restricted segmental motion in BCB thermosets are generally associated with higher *T*_g_ values and lower CTEs.^37,38^ In line with these findings, Cured BCB-B, which contains a rigid biphenyl bridge, exhibited the highest *T*_g_ (329.1 °C) and the lowest CTE (23.8 ppm °C^−1^). This combination reflects the limited rotational freedom imparted by its biphenyl structure. By contrast, the lower *T*_g_ values of Cured BCB-A and Cured BCB-F (287.5 and 289.5 °C, respectively) reflect the comparatively greater conformational freedom conferred by their isopropylidene and methylene bridges. The relatively high *T*_g_ of Cured BCB-AF (313.0 °C) is attributed to the restricted segmental motion imposed by its bulky hexafluoroisopropylidene bridge, in agreement with the reported behavior of related BCB-containing aromatic polymers.^39,40^

**Fig. 6:**
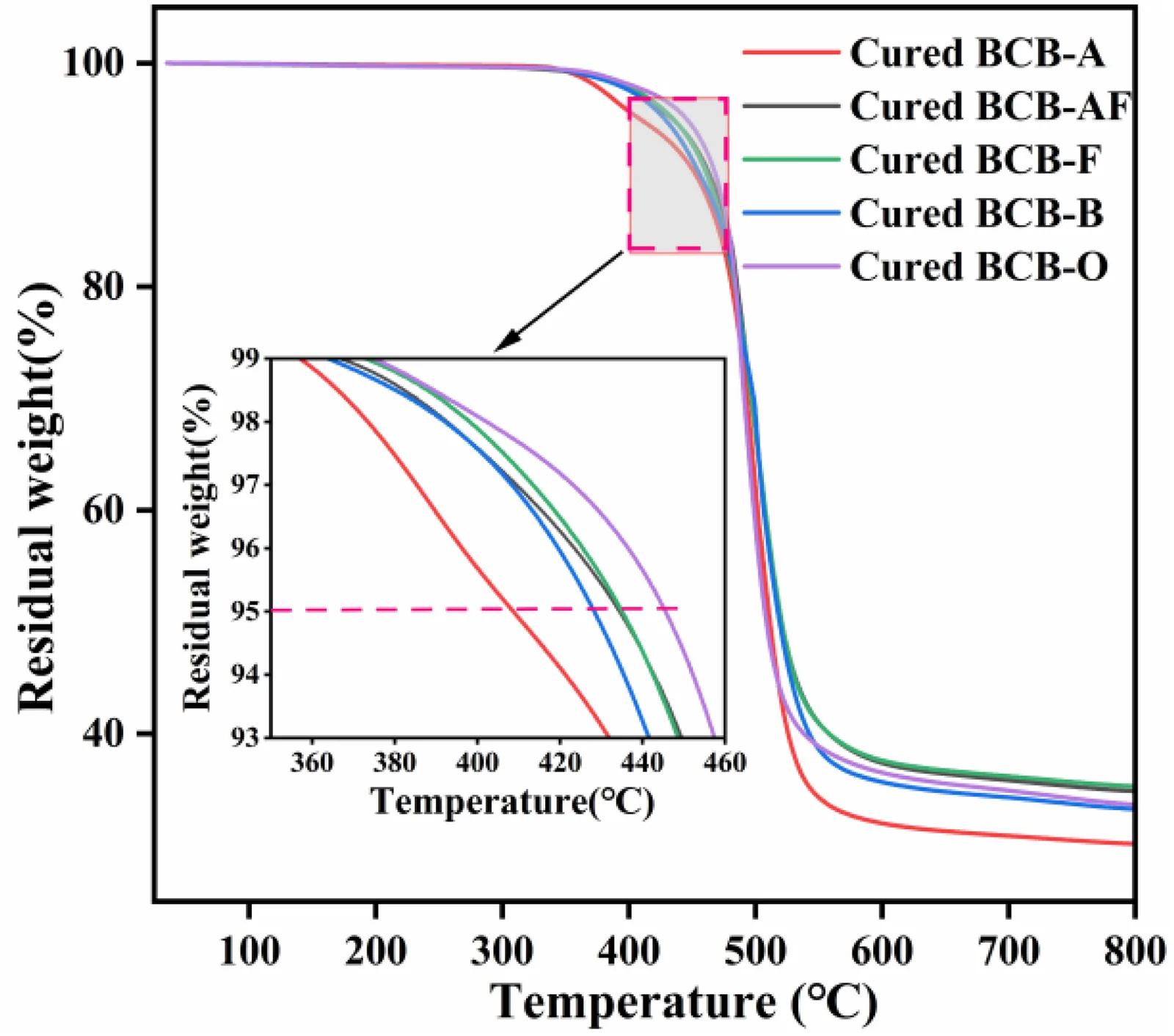
TGA curves of the cured BCB resins at a heating rate of 10 °C min^−1^ under N_2_.

The thermal degradation temperatures of the five samples under a nitrogen atmosphere were assessed by TGA. As shown in Fig. 6, all cured resins exhibited high 5% weight-loss temperatures (*T*_5%_) ranging from 408 to 445 °C. The maximum degradation temperatures (*T*_peak_) of the cured samples were in the range of 487 °C to 503 °C, with residual weights of 30–35% at 800 °C. These values are superior to those of many reported BCB-based polymers,^28,41^ indicating that this series of polymers possesses excellent thermal stability.

### 3.4 Mechanical properties

**Fig. 7:**
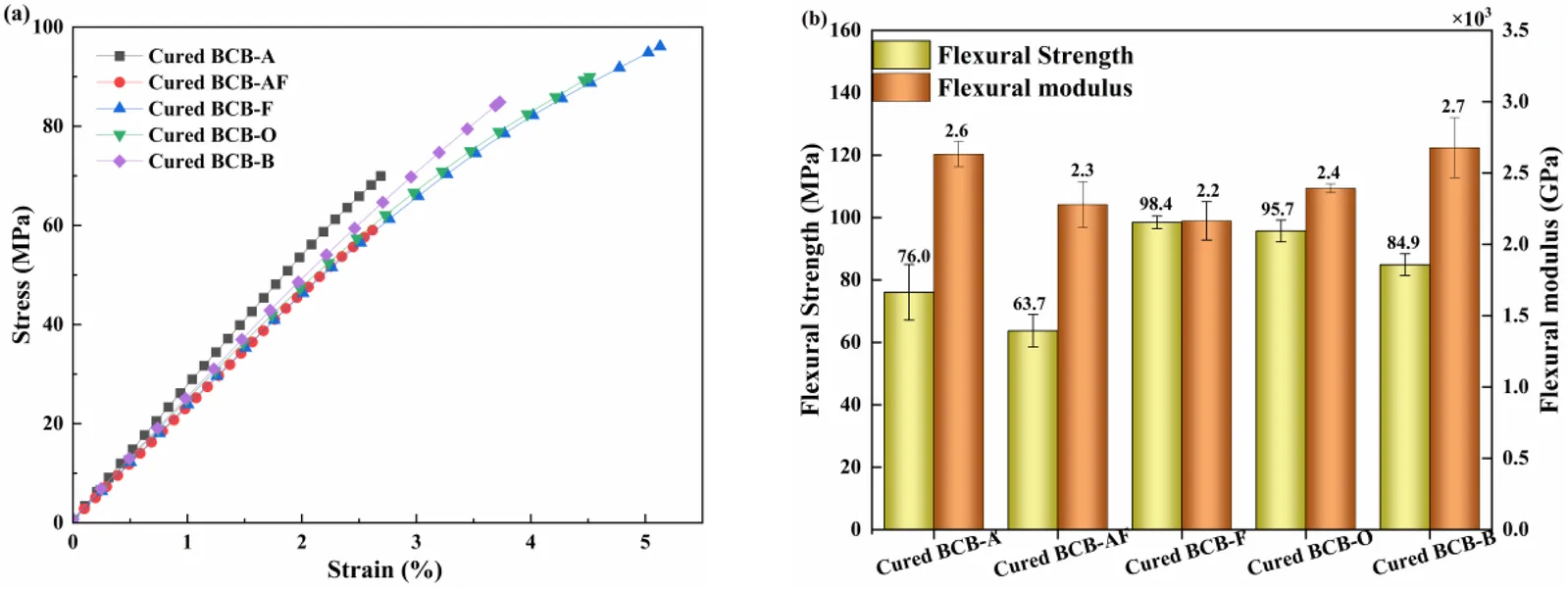
Flexural properties of the cured BCB resins: (a) stress–strain curves and (b) flexural strength and modulus.

To further evaluate the mechanical performance of the cured BCB resins, their flexural properties were investigated by three-point bending tests. The flexural properties of the cured BCB resins are shown in Fig. 7. The higher strains and greater toughness of Cured BCB-F and Cured BCB-O are attributed to the low steric hindrance of their methylene and ether (–O–) linkages, respectively. The flexural strengths of Cured BCB-A, Cured BCB-AF, Cured BCB-F, Cured BCB-O, and Cured BCB-B were 76.0, 63.7, 98.4, 95.7, and 84.9 MPa, respectively, while the corresponding flexural moduli were 2.6, 2.3, 2.2, 2.4, and 2.7 GPa. Among them, Cured BCB-F exhibited the highest flexural strength of 98 MPa, whereas Cured BCB-B showed the highest flexural modulus of 2.7 GPa. These values are comparable to those reported for fluorinated BCB resins, which exhibited flexural strengths of 68–88 MPa and flexural moduli of 2.52–3.15 GPa.^42^ Moreover, a recently reported low-dielectric polybenzoxazine exhibited a flexural strength of 82.6 MPa and a flexural modulus of 2.2 GPa.^43^ Therefore, the present cured BCB resins exhibit good flexural mechanical properties.

### 3.5 Hydrophobicity

**Fig. 8:**
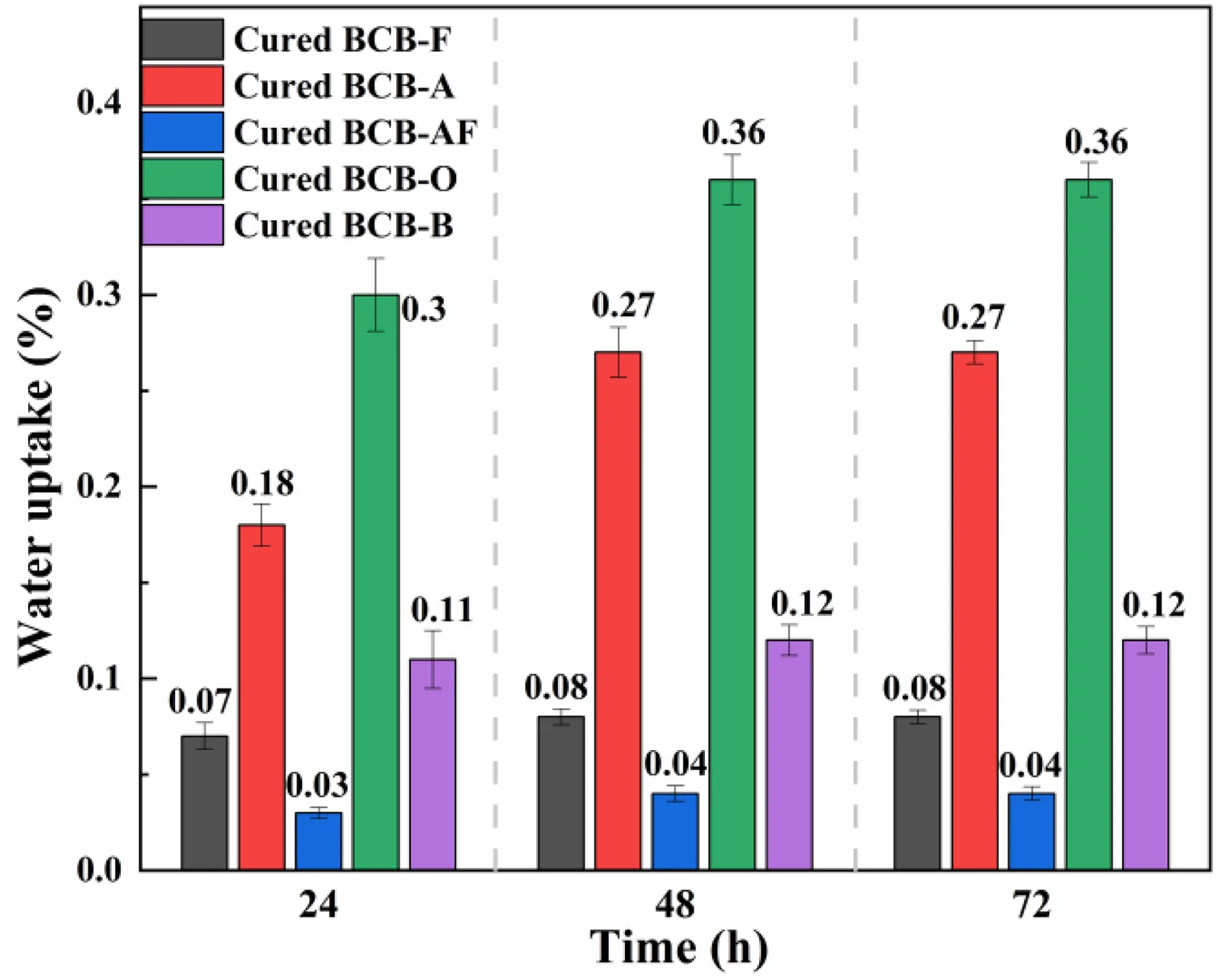
Water uptake of the cured samples after immersion in boiling water.

For low-dielectric-constant materials, good hydrophobicity and low water uptake are essential, as moisture can significantly impair the dielectric performance of devices in practical applications. To evaluate the water uptake of the samples, immersion tests were carried out, and the results are shown in Fig. 8. Boiling-water immersion was employed as an accelerated and stringent hygrothermal test to compare the water resistance of the cured resins under the combined effects of high temperature and direct water contact. The water uptake changed only slightly from 24 to 48 h and approached a plateau thereafter, indicating near-steady water uptake under the present boiling-water immersion conditions. Among the five cured resins, Cured BCB-AF exhibited the lowest water uptake of 0.04% after 72 h, whereas Cured BCB-O showed the highest value of 0.36%. The low water uptake of Cured BCB-AF is consistent with the presence of hydrophobic fluorinated units, particularly the –CF_3_ groups. Nevertheless, the long-term stability of the dielectric and thermomechanical properties under prolonged thermal aging, controlled humidity exposure and repeated thermal cycling remains to be systematically evaluated in future work.

### 3.6 Dielectric properties of cured dibenzocyclobutene resins

Dielectric constant (*D*_k_) and dissipation factor (*D*_f_) are key parameters for evaluating the dielectric performance of materials. For high-frequency communication applications, low *D*_k_ and *D*_f_ are desirable for maintaining signal transmission speed and minimizing signal delay or loss. In this study, the *D*_k_ and *D*_f_ of the five cured dibenzocyclobutene resins were measured at 5 and 10 GHz, as summarized in Table 3. The dielectric properties of the cured BCB resins were evaluated at both 5 and 10 GHz, and the results are summarized in Table 3. At 10 GHz, the *D*_k_ values of Cured BCB-A, Cured BCB-AF, Cured BCB-F, Cured BCB-B, and Cured BCB-O were 2.69, 2.54, 2.67, 2.80, and 2.83, respectively, while their corresponding *D*_f_ values were 2.76 × 10^−3^, 2.32 × 10^−3^, 3.63 × 10^−3^, 2.90 × 10^−3^, and 3.31 × 10^−3^. At 5 GHz, the *D*_k_ values were 2.64, 2.52, 2.62, 2.36, and 2.71, respectively, and the corresponding *D*_f_ values were 2.54 × 10^−3^, 2.10 × 10^−3^, 3.62 × 10^−3^, 2.35 × 10^−3^, and 3.19 × 10^−3^. These results demonstrate that all cured BCB resins maintained low dielectric constants and low dielectric losses in the investigated frequency range of 5–10 GHz. The dielectric properties after moisture exposure were further evaluated at 5 and 10 GHz. After moisture absorption, the *D*_k_ values ranged from 2.56 to 2.82 at 5 GHz and from 2.55 to 2.87 at 10 GHz, while the corresponding *D*_f_ values increased to 3.16 × 10^−3^ to 5.71 × 10^−3^ and 3.46 × 10^−3^ to 6.47 × 10^−3^, respectively. Compared with the initial state, moisture exposure mainly increased the dielectric loss of the cured resins, which was associated with the introduction of absorbed water molecules. Among the five resins, Cured BCB-AF exhibited the lowest *D*_f_ after moisture exposure at both frequencies, consistent with its low water uptake and the hydrophobic fluorinated structure. In contrast, Cured BCB-O showed a relatively higher *D*_f_ after moisture exposure, corresponding to its higher water uptake.

**Table 3:** Dielectric properties of BCB-based resins containing C–O linkages

Resin	Structure of precursors	*D*_k_	*D*_f_ (10^−3^)	Frequency (GHz)	Ref.
Cured BCB-F	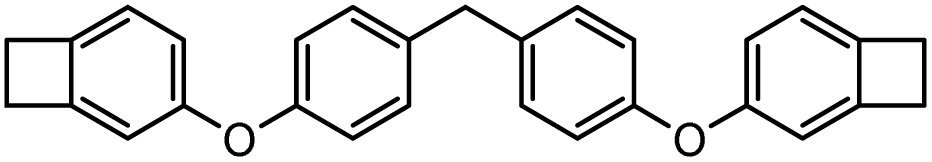	2.67	3.63	10	This work
		2.77^a^	6.09^a^		
		2.62	3.62	5	
		2.77^a^	5.71^a^		
Cured BCB-A	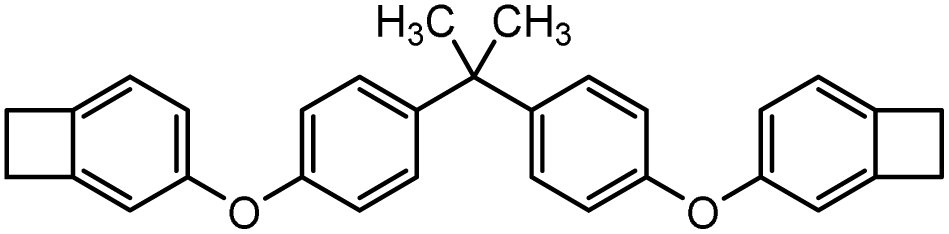	2.69	2.76	10	This work
		2.70^a^	4.26^a^		
		2.64	2.54	5	
		2.71^a^	3.87^a^		
Cured BCB-AF	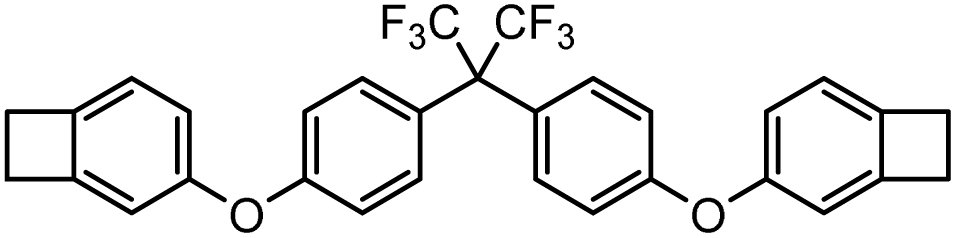	2.54	2.32	10	This work
		2.55^a^	3.46^a^		
		2.52	2.10	5	
		2.56^a^	3.16^a^		
Cured BCB-B	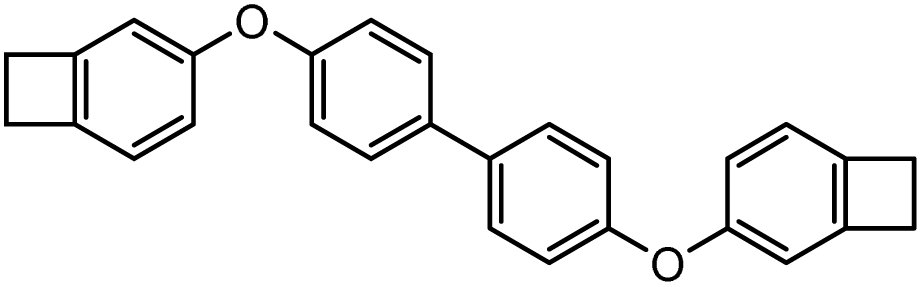	2.80	2.90	10	This work
		2.82^a^	5.41^a^		
		2.36	2.35	5	
		2.82^a^	4.76^a^		
Cured BCB-O	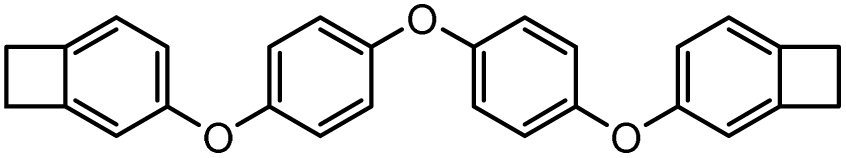	2.83	3.31	10	This work
		2.87^a^	6.47^a^		
		2.71	3.19	5	
		2.79^a^	5.61^a^		
M3	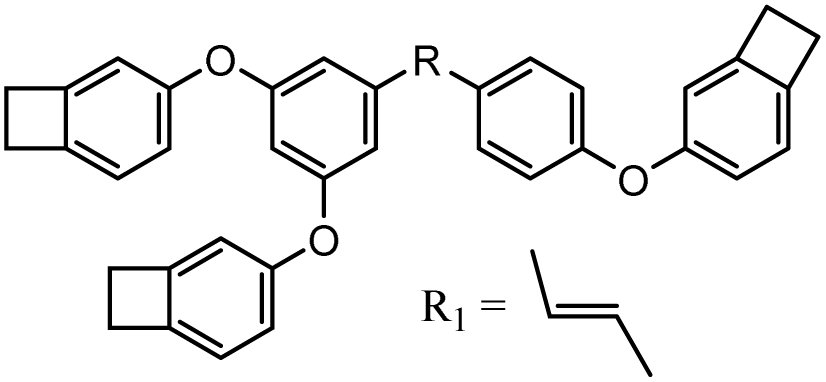	2.84	7.0	5	22
M4	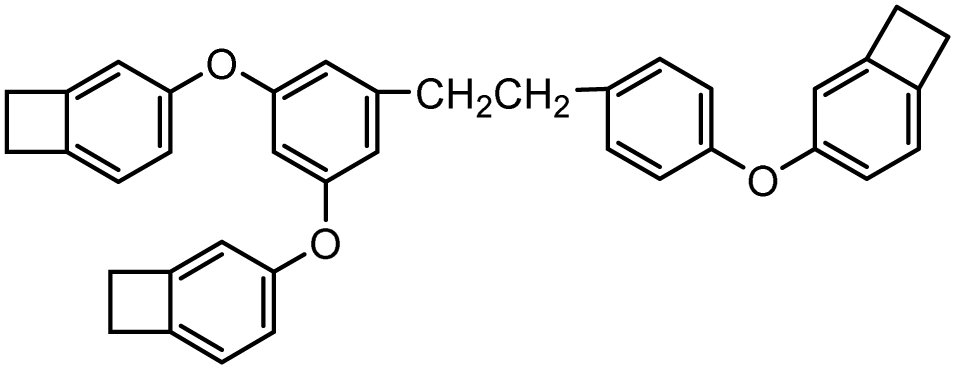	2.81	3.2	5	22
DA-BCB	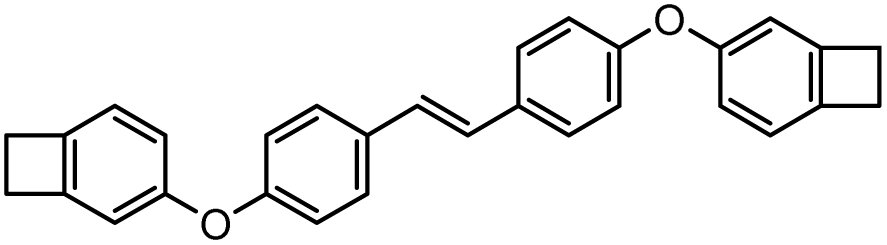	2.73	2.44	10	24
DI-BCB	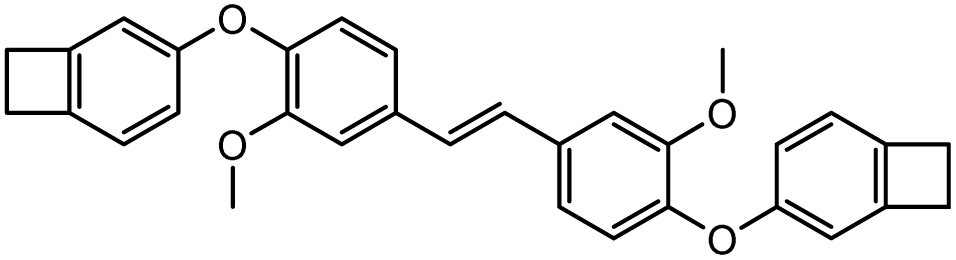	3.04	11.8	10	24
MC	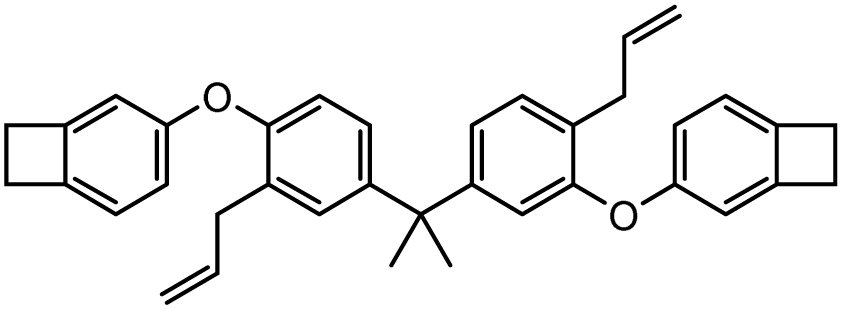	2.67	—	5	45
DCPDNO-BCB	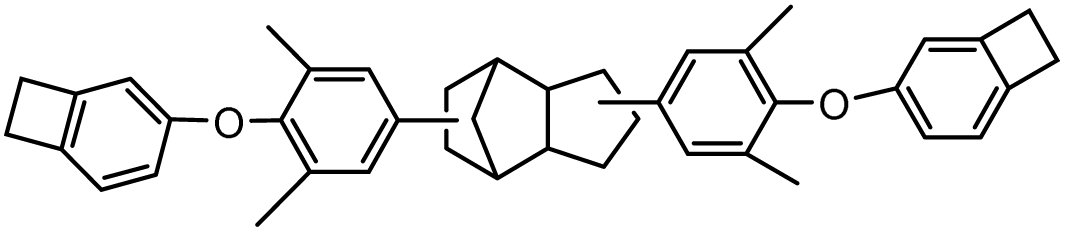	2.53	3.97	0.01	29
Bio-based M1	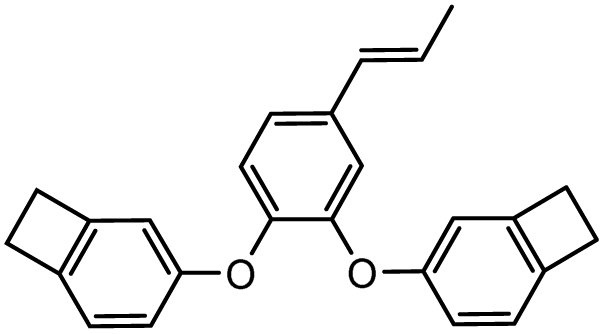	2.59	5.36	5	26
AB	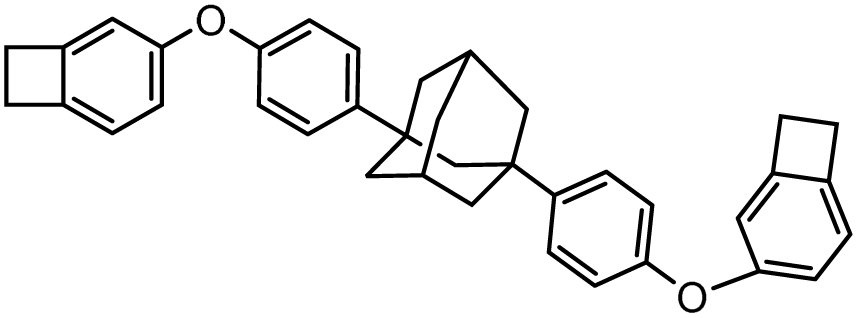	2.71	2.67	10	28
PBBCB-O-C6	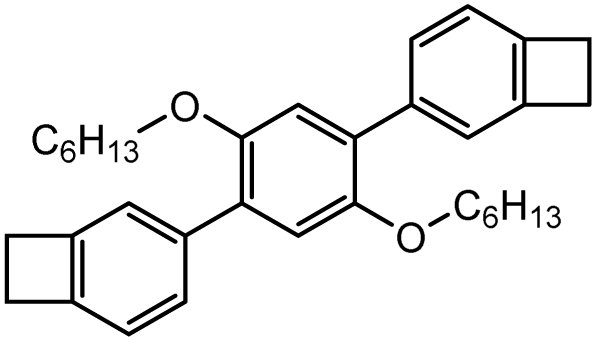	2.64	10.8	10	36
4F-bis-BCB	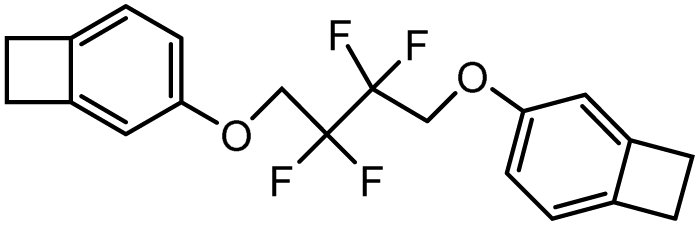	2.73	—	10	46
C-BPF	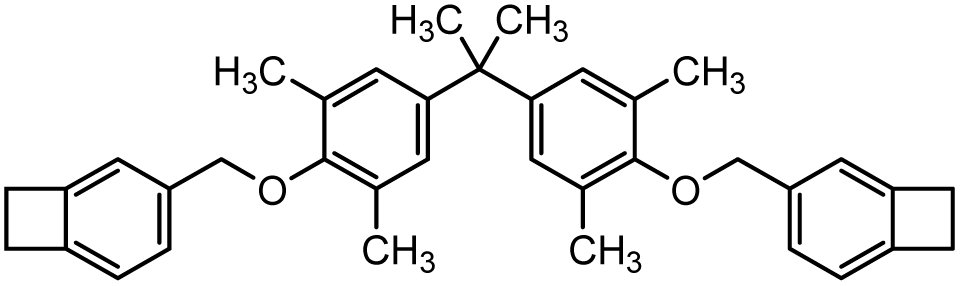	2.65	7.2	10	This work (see SI)

a Measured after the resins were immersed in boiling water for 72 h.

Structurally, all resins contain C–O bonds (*e.g.*, aryl ether linkages), which exhibit low polarity and lower polarizability than strongly polar groups such as C=O or O–H, thereby contributing favorably to a low *D*_k_. This observation aligns with the established principle that low polarizability groups (*e.g.*, C–F, C–O, C–C, C–H) help reduce *D*_k_.^44^

The dissipation factor (*D*_f_) is sensitive to subtle variations in molecular structure. *D*_f_ primarily reflects the energy dissipation caused by the orientational motion of dipoles under an alternating electric field, and is strongly influenced by chain rigidity and segmental mobility.^47^ The structural differences among the five BCB resins primarily arise from the varied linking groups. These results suggest that low-polarity moieties, such as methyl (–CH_3_) and trifluoromethyl (–CF_3_) groups, contribute to a reduction in *D*_f_. In particular, the presence of fluorinated groups (–CF_3_) enables BCB-AF to exhibit the lowest *D*_f_ of 2.32 × 10^−3^ among the series of dibenzocyclobutene resins.

**Fig. 9:**
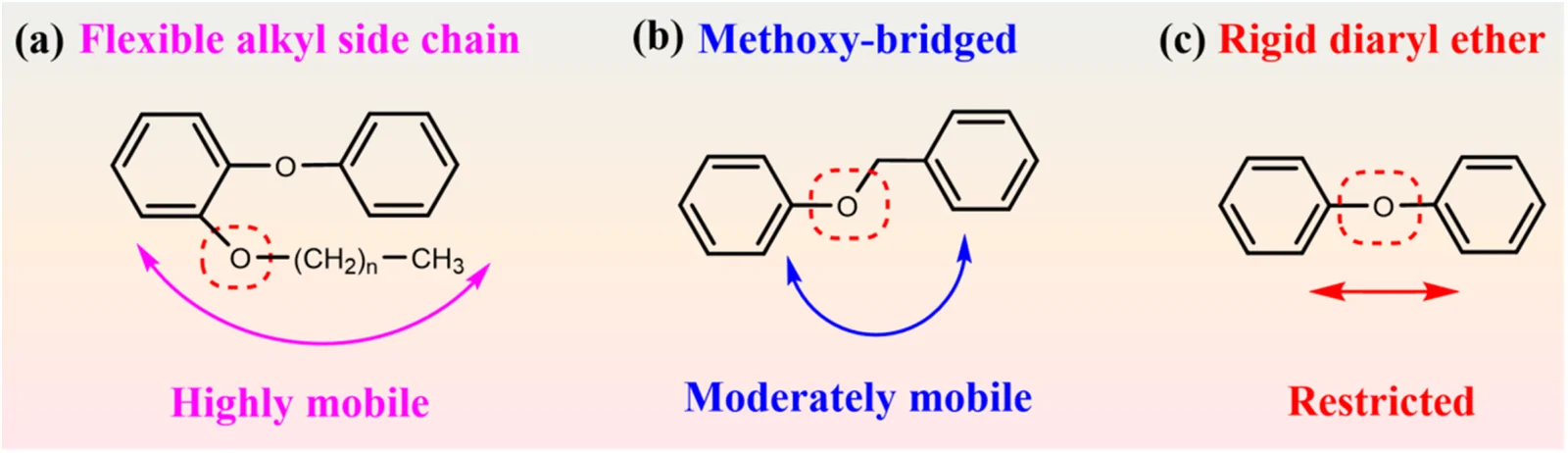
Schematic representation of three types of C–O linkages.

Table 3 also compares reported BCB resins containing different C–O linkages, indicating that the chemical environment of the C–O linkage is associated with differences in *D*_f_. When the C–O bond connects a benzene ring to a flexible alkyl chain (see 9(a) and 9(b) in Fig. 9), the resins exhibit relatively high *D*_f_ values, as exemplified by DI-BCB (Ph–O–CH_3_, *D*_f_ = 11.8 × 10^−3^),^24^PBBCB-O-C6 (Ph–O–C_6_H_13_, *D*_f_ = 10.8 × 10^−3^),^36^ and C-BPF (Ph–O–CH_2_–Ph, *D*_f_ = 7.2 × 10^−3^) (Fig. S14 and S15). This behavior can be rationalized by Debye's theory of dielectric relaxation: enhanced molecular chain mobility increases the orientational polarization contribution of dipoles, thereby raising *D*_f_.^47^ In contrast, in each of the five resins developed in this work, the C–O bond is embedded within a rigid aromatic main chain (Ph–O–Ph, see 9(c) in Fig. 9). The enhanced rigidity of the aromatic main chain restricts dipole motion and thereby contributes to the low *D*_f_ values observed.

## 4 Conclusions

In this study, five dibenzocyclobutene resins (BCB-A, BCB-AF, BCB-F, BCB-B, and BCB-O) were synthesized from commercially available, low-cost bisphenols *via* a facile Ullmann reaction. The reaction employed CuI as the catalyst, 1-butylimidazole as the ligand, K_2_CO_3_ as the base, and 4-bromobenzocyclobutene as both the reactant and the solvent. This synthetic route avoids expensive Cs_2_CO_3_ and oxalamide ligands, significantly reducing the resin cost and offering great potential for industrial application. All resins exhibited appropriate melting points (59–120 °C) and a wide curing window, with negligible mass loss during curing, demonstrating excellent processability. After thermal curing, the resulting polymers showed *T*_g_ values of 287–329 °C, *T*_5%_ values of 408–445 °C, and CTE values of 23.8–62.8 ppm °C^−1^. The cured BCB resins exhibited flexural strengths of 63.7–98.4 MPa and flexural moduli of 2.2–2.7 GPa, demonstrating their good mechanical integrity. The resins displayed low water uptake of 0.04–0.36% after immersion in boiling water for 72 h, reflecting good hydrophobicity. At 10 GHz, all cured resins exhibited *D*_k_ below 2.83 and *D*_f_ lower than 3.63 × 10^−3^, with the fluorine-containing Cured BCB-AF showing the lowest *D*_f_ of 2.32 × 10^−3^. After moisture exposure, the resins maintained low dielectric constants and losses, while Cured BCB-AF showed the lowest dielectric loss due to its low water uptake and hydrophobic fluorinated structure. Collectively, the bisphenol-based dibenzocyclobutene resins developed in this work combine low raw-material and synthesis costs, excellent thermal properties, favorable high-frequency dielectric performance and strong hydrophobicity, demonstrating their potential for further evaluation as dielectric resins for high-frequency communication applications.

## Author contributions

Honghui Cheng and Lei Wu: data curation (lead), formal analysis (lead), investigation (lead), writing – original draft (lead). Jiafeng Di: investigation (supporting), methodology (supporting), validation (lead), writing – original draft (supporting). Hua Lai: funding acquisition (lead), project administration (lead), resources (lead), writing–review and editing (lead). Yanyu Li: data curation (supporting), investigation (lead), methodology (supporting), writing – review and editing (supporting). Chi Zhang: funding acquisition (lead), project administration (lead), resources (lead), supervision (lead). Xiao Wu: methodology (supporting), supervision (supporting). JinRong Ye: investigation (supporting), methodology (supporting).

## Conflicts of interest

The authors declare no conflicts of interest.

## Supplementary Material

RA-OLF-D6RA06919C-s001

## Data Availability

The data that support the findings of this study are available from the corresponding author upon reasonable request. Supplementary information (SI): synthesis procedures of C-BPF and mono-substituted BPF-BCB; ^1^H NMR, ^13^C NMR, and ^19^F NMR spectra, and cost calculations of the synthesized BCB resins. See DOI: https://doi.org/10.1039/d6ra06919c.
