# Supplementary material for: Economical bisphenol-based dibenzocyclobutene resins with excellent thermal and dielectric properties for high-frequency communication applications
Source: RSC Adv. 2026 Sep 7;16(43):53112–22. doi: 10.1039/d6ra06919c (PMC13548190; doi:10.1039/d6ra06919c)
Supplement: Supplementary file 1 [file RA-016-D6RA06919C-s001.pdf]

Supplementary Information (SI) for RSC Advances.
This journal is © The Royal Society of Chemistry 2026

Supporting Information for

Economical bisphenol-based dibenzocyclobutene resins with excellent thermal and dielectric properties for high-frequency communication applications

Honghui Cheng,[†a] Lei Wu,[†a] JiaFeng Di,[a] Hua Lai,*[a] Yanyu Li,*[b] Chi Zhang,[b] Xiao Wu,[b] JinRong Ye[a]

[a] *College of Chemistry and Materials Science, Hengyang Normal University, Hengyang, China. E-mail: laixhua163@163.com*

[b] *Tongyu Advanced Materials (Guangdong) CO., LTD, Zhaoqing, China. E-mail: yanyu_li@tyadmt.com*

[†] *These authors contributed equally to this work.*

Contents:

·EXPERIMENTAL SECTION

Synthesis of mono-substituted BPF-BCB intermediate according to Ma et al.'s procedure[1, 2]

Synthesis procedure of C-BPF

·CHARACTERIZATIONS

Table S1 Estimate laboratory-scale raw-material costs of representative BCB resins

Table S2 Weight loss of the five resins before and after curing

Fig. S1 ^{1}H NMR spectrum of mono-BCB prepared from BPF following Ma Dawei's procedure (500 MHz, $CDCl_3$).

Fig. S2 ^{1}H NMR spectrum of the monomers **BCB-AF** (500 MHz, $CDCl_3$).

Fig. S3 ^{1}H NMR spectrum of the monomers **BCB-A** (500 MHz, $CDCl_3$).

Fig. S4 ^{1}H NMR spectrum of the monomers **BCB-F** (500 MHz, $CDCl_3$).

Fig. S5 ^{1}H NMR spectrum of the monomers **BCB-O** (500 MHz, $CDCl_3$).

Fig. S6 ^{1}H NMR spectrum of the monomers **BCB-B** (500 MHz, $CDCl_3$).

Fig. S7 ^{13}C NMR spectrum of the monomers **BCB-AF** (126 MHz, $CDCl_3$).

Fig. S8 ^{13}C NMR spectrum of the monomers **BCB-A** (126 MHz, $CDCl_3$).

Fig. S9 ^{13}C NMR spectrum of the monomers **BCB-F** (126 MHz, $CDCl_3$).

Fig. S10 ^{13}C NMR spectrum of the monomers **BCB-O** (126 MHz, $CDCl_3$).

Fig. S11 ^{13}C NMR spectrum of the monomers **BCB-B** (126 MHz, $CDCl_3$).

Fig. S12 ^{19}F NMR spectrum of the monomers **BCB-AF** (471 MHz, $CDCl_3$).

Fig. S13 FT-IR spectra of samples of **BCB-AF** precured at different temperatures for 1h.

Fig. S14 ^{1}H NMR spectrum of the monomers **C-BPF** (500 MHz, $CDCl_3$).

Fig. S15 ^{13}C NMR spectrum of the monomers **C-BPF** (126 MHz, $CDCl_3$).

EXPERIMENTAL SECTION

Synthesis of mono-substituted BPF-BCB intermediate according to Ma et al.'s procedure[1,2]

4-Bromobenzocyclobutene, bisphenol F, copper(I) iodide, cesium carbonate, and Ligand L are added to a dry three-necked round-bottom flask that has been purged with argon, and DMF is used as the solvent. The mixture is heated to 140 °C and stirred for 16 h. After cooling to room temperature, the mixture is filtered. The organic phase is washed with water, dried over anhydrous Na_2SO_4, and concentrated under reduced pressure. The residue is purified by column chromatography to afford the product as a crystalline powder.

Synthesis procedure of C-BPF

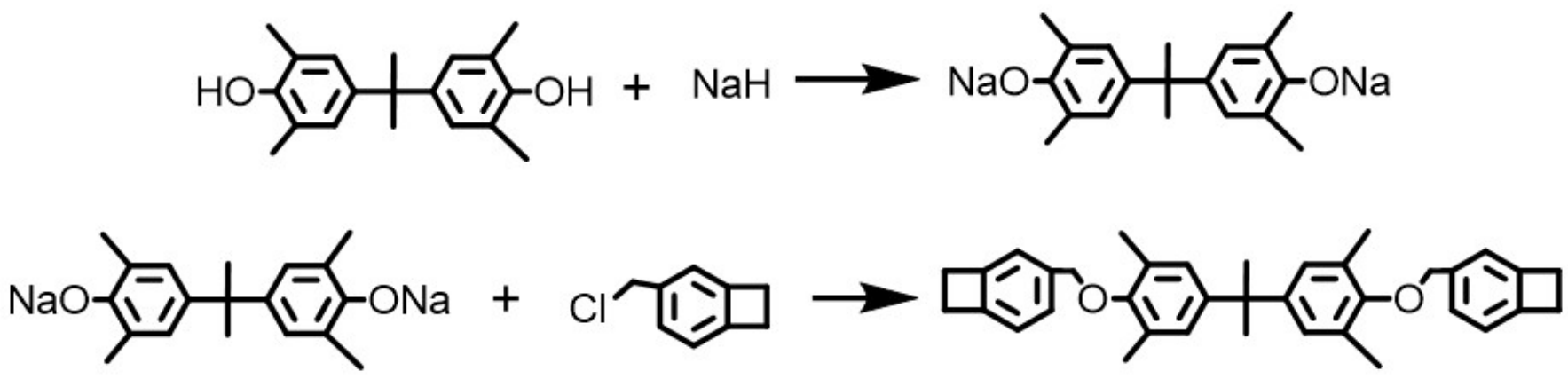

The synthesis is carried out in a dry three-necked flask under an argon atmosphere using DMF as the solvent. 2,6-Dimethylphenol (TMBPA) is reacted with NaH, and the reaction progress is monitored by thin-layer chromatography (TLC). After the starting material has been consumed, 4-(chloromethyl)benzocyclobutene (4-Cl-BCB) is added slowly via a dropping funnel. Upon completion of the reaction, the mixture is filtered through a Büchner funnel, and the collected solid is washed with deionized water. The solid is then recrystallized from ethanol to afford the desired product as white needle-like crystals in 84.2% yield.

CHARACTERIZATIONS

Table S1 Estimation of costs of representative BCB resins

Resin	Bisphenol/Core precursor / RMB·mol^{-1}	Ligand /Additive/ RMB·mol^{-1}	Catalyst / RMB·mol^{-1}	Base / RMB·mol^{-1}	4-BrBCB / RMB·mol^{-1}	Solvent / RMB·mol^{-1}	Yield/ %	Total / RMB·mol^{-1}	Total / RMB·g^{-1}
**BCB-A**	BPA: 2.28	1-Butylimidazole: 8.38	CuI: 395.37	K_2CO_3: 1.59	549.15	0	65.6	1189.62	2.75
**BCB-AF**	BPAF: 23.54	1-Butylimidazole: 8.38	CuI: 395.37	K_2CO_3: 1.59	549.15	0	67.8	1210.88	2.24
**BCB-F**	BPF: 13.22	1-Butylimidazole: 8.38	CuI: 395.37	K_2CO_3: 1.59	549.15	0	66.1	1200.56	2.97
**BCB-O**	BPO: 13.35	1-Butylimidazole: 8.38	CuI: 395.37	K_2CO_3: 1.59	549.15	0	55.8	1200.69	2.95
**BCB-B**	BPB: 9.31	1-Butylimidazole: 8.38	CuI: 395.37	K_2CO_3: 1.59	549.15	0	53.18	1196.65	3.06
**AB**[2]	M1: 676	Ligand L: 91	CuI: 395.37	$C_{S2}CO_3$: 847.13	549.15	DMF: 8.09	49.0	12027.91	22.92
**DCPDNO-BCB**[3]	DCPDNO: 892.69	DPM: 37.28	CuCl (>99.95%): 514.38	$C_{S2}CO_3$: 847.13	549.15	NMP: 12.43	35.0	15559.40	26.82

*Reagent prices were obtained from official commercial supplier quotations; the costs of the five resins developed in this work were calculated from their actual experimental consumption, whereas those of **AB** and **DCPDNO-BCB** were calculated according to the corresponding published synthetic procedures.

Table S2. Weight loss of five resins during curing

Resins	Weight Change 1 (%)	Weight Change 2 (%)
**BCB-A**	10.2	0.20
**BCB-F**	0.5	0.08
**BCB-AF**	3.7	0.12
**BCB-B**	15.3	0.60
**BCB-O**	12.6	0.10

(In the curing process of dibenzocyclobutene resins, **Weight Change 1** is defined as the weight loss rate during curing under vacuum conditions, whereas **Weight Change 2** is defined as the weight loss rate during curing under 1 atm in a nitrogen atmosphere.)

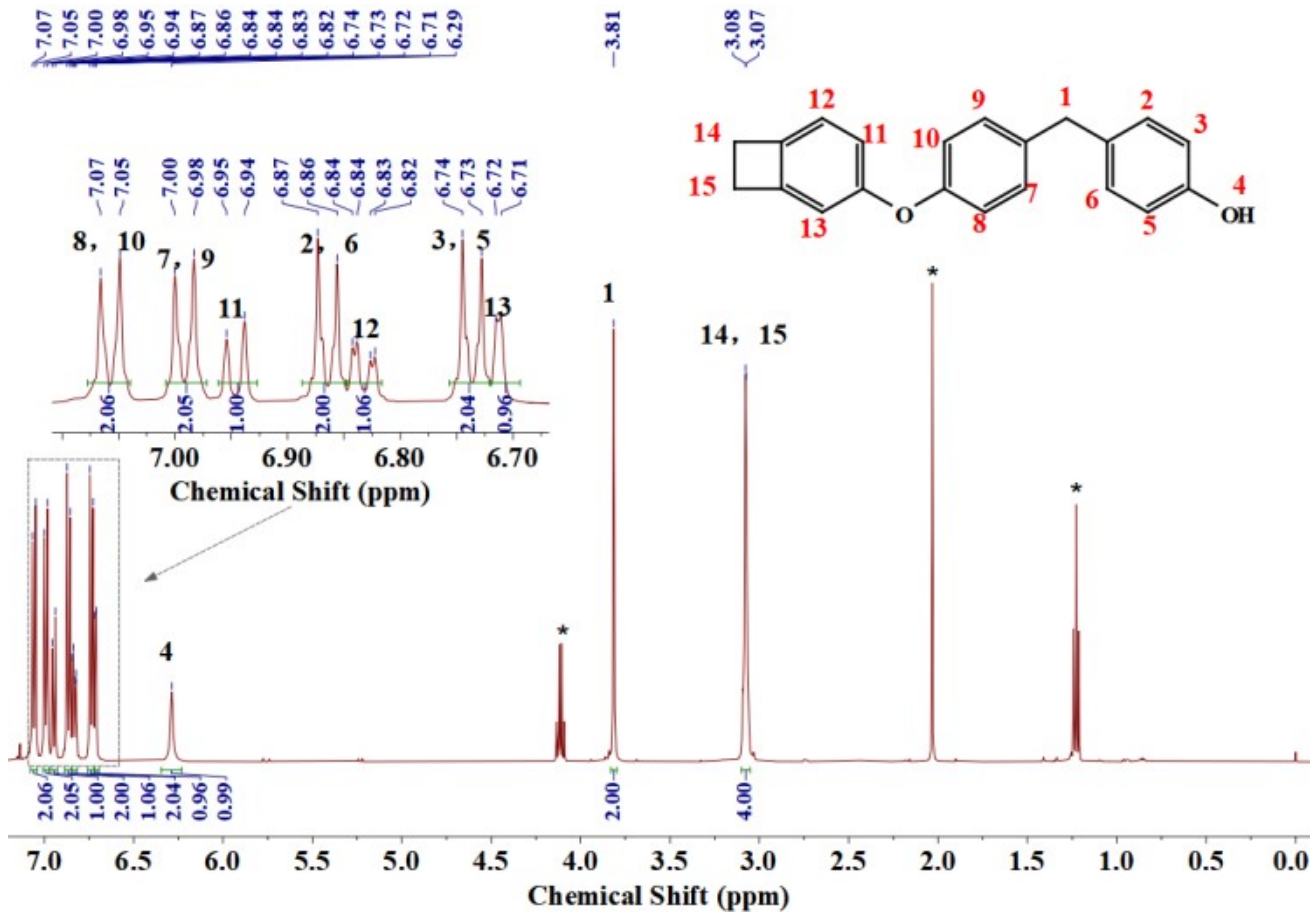

Fig. S1 ^{1}H NMR spectrum of mono-BCB prepared from BPF following Ma Dawei's procedure(500 MHz, $CDCl_3$).
(Note: The peaks marked with an asterisk (*) correspond to the solvent peak of ethyl acetate)

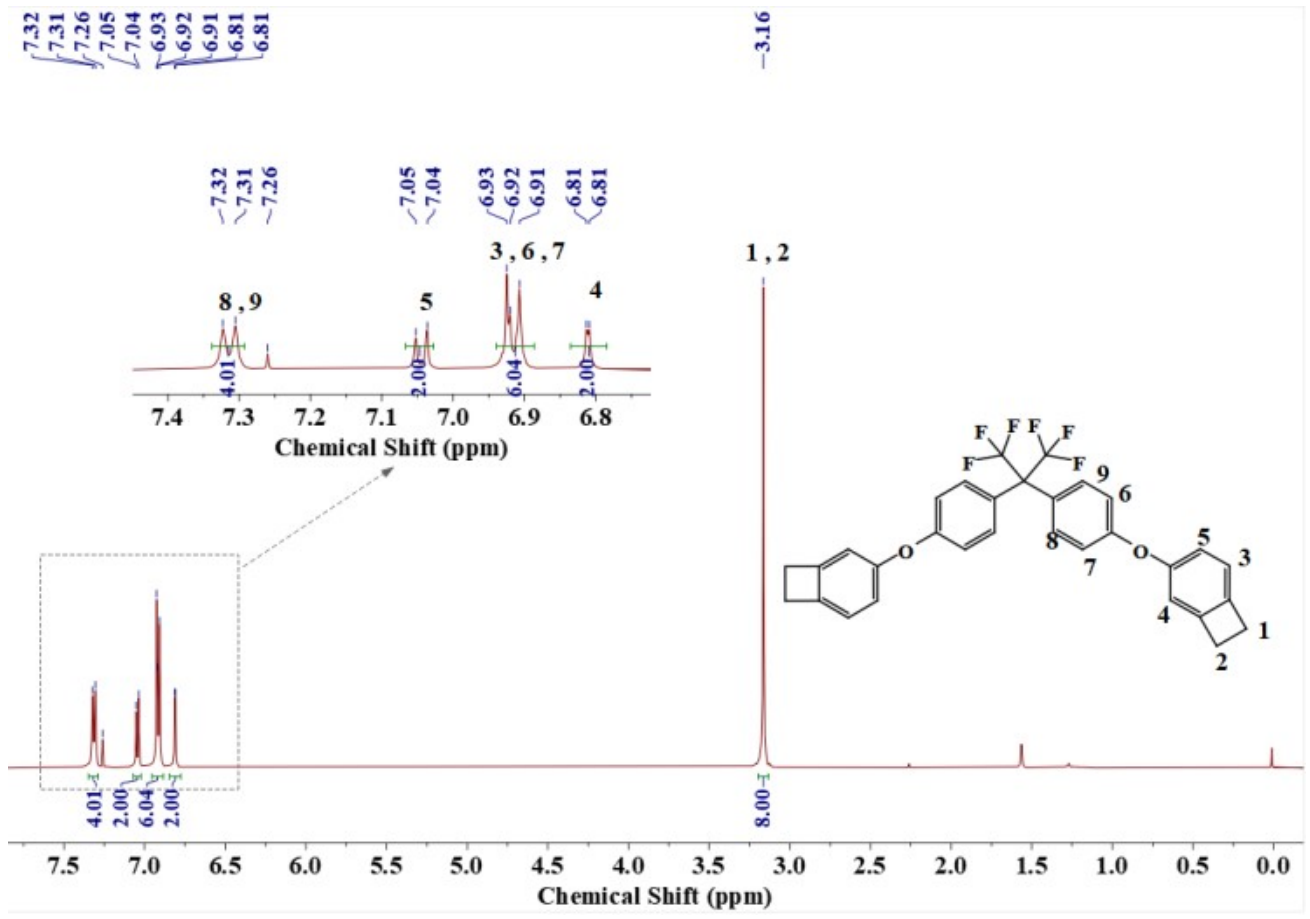

Fig. S2. ^{1}H NMR spectrum of the monomers **BCB-AF** (500 MHz, $CDCl_3$).

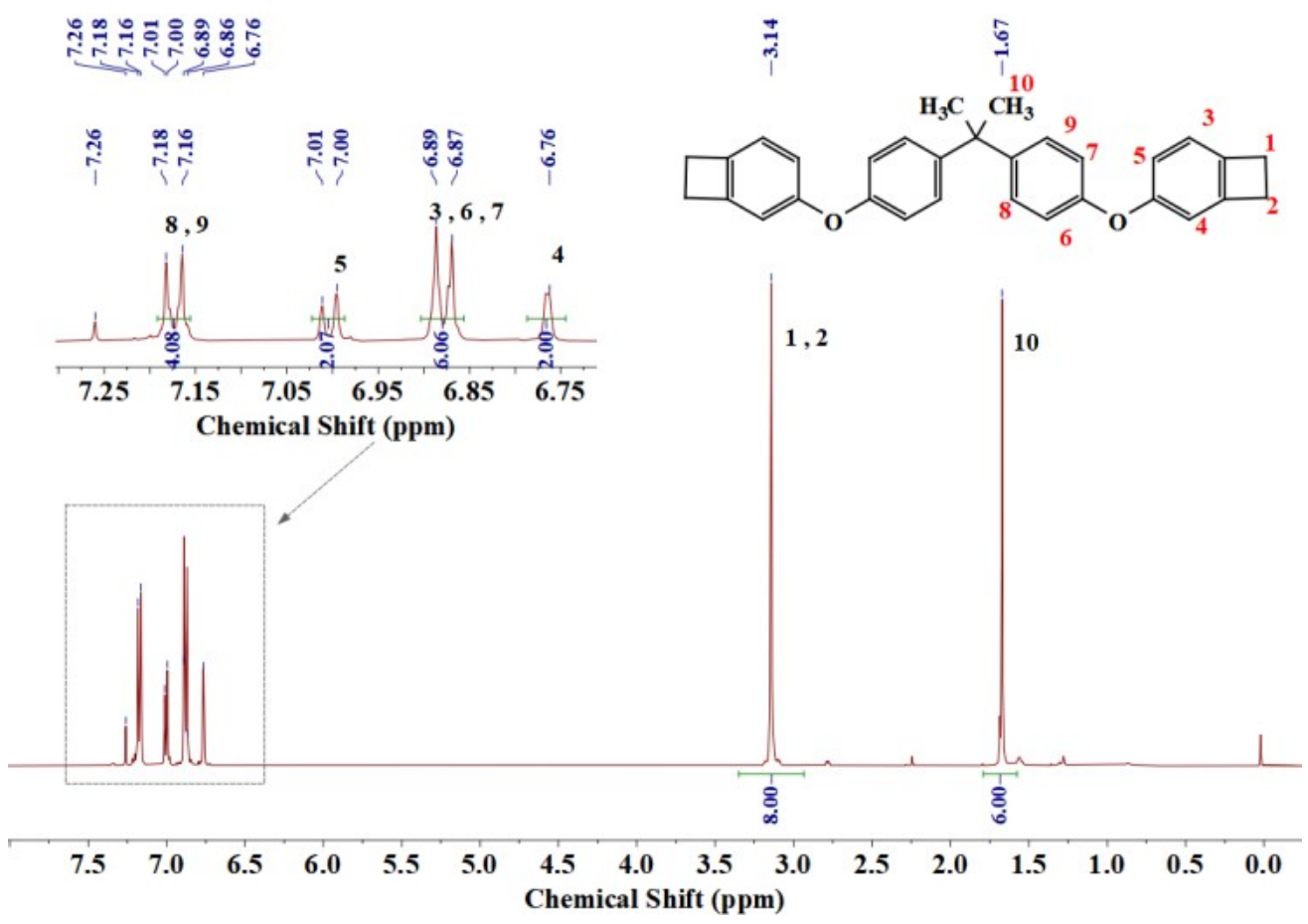

Fig. S3. ^{1}H NMR spectrum of the monomers **BCB-A** (500 MHz, $CDCl_3$).

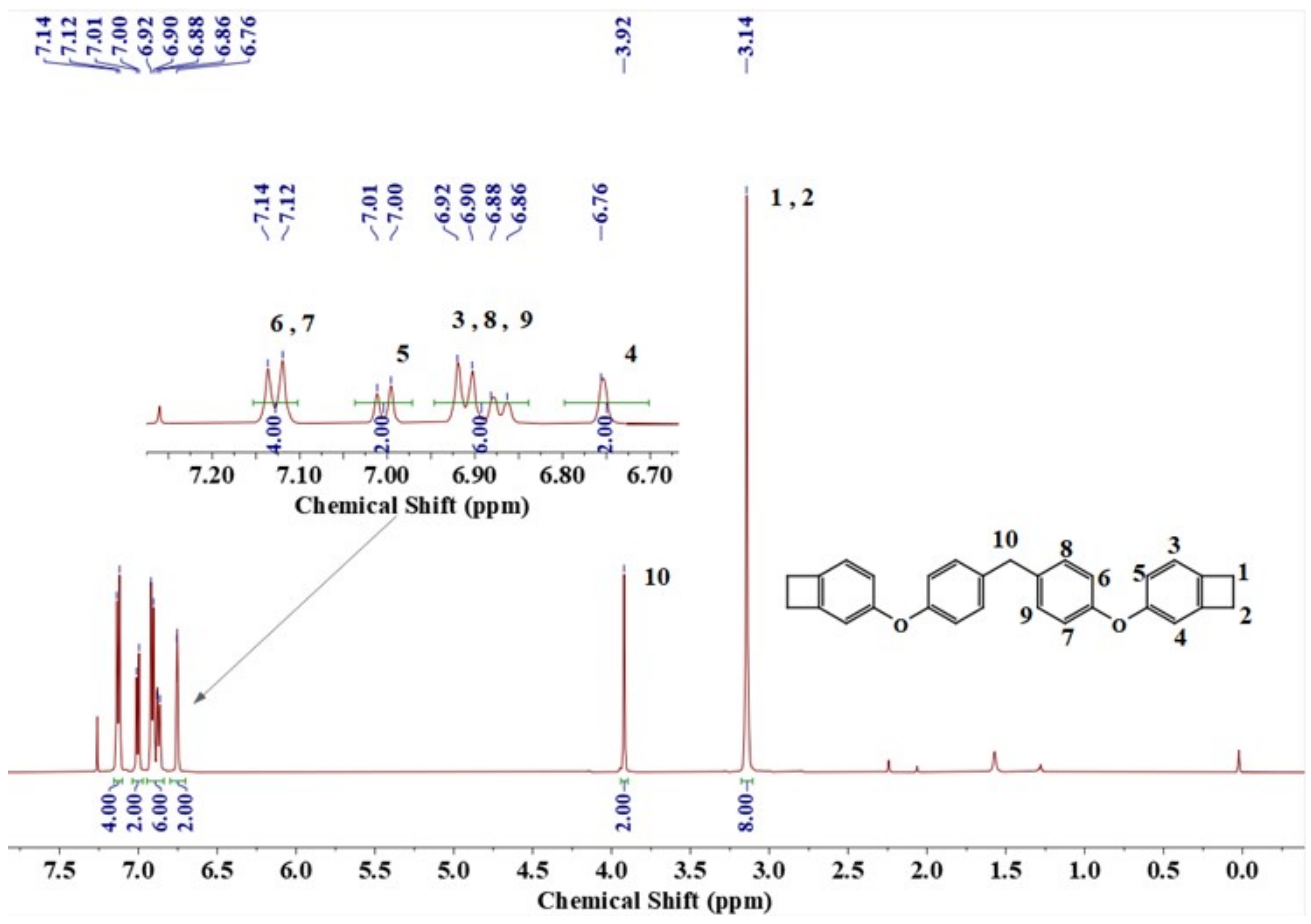

Fig. S4. ^{1}H NMR spectrum of the monomers **BCB-F** (500 MHz, $CDCl_3$).

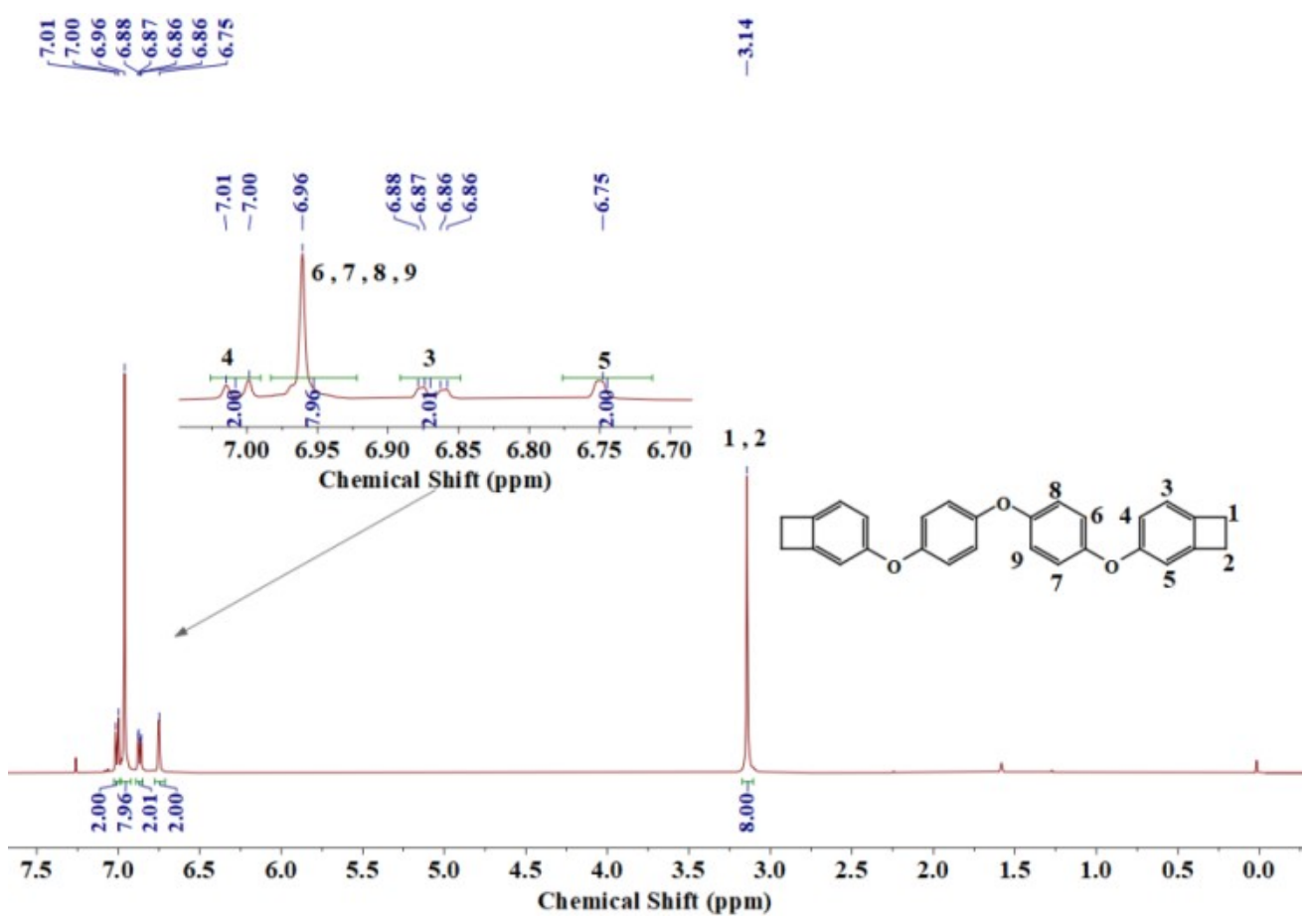

Fig. S5. ^{1}H NMR spectrum of the monomers **BCB-O** (500 MHz, $CDCl_3$).

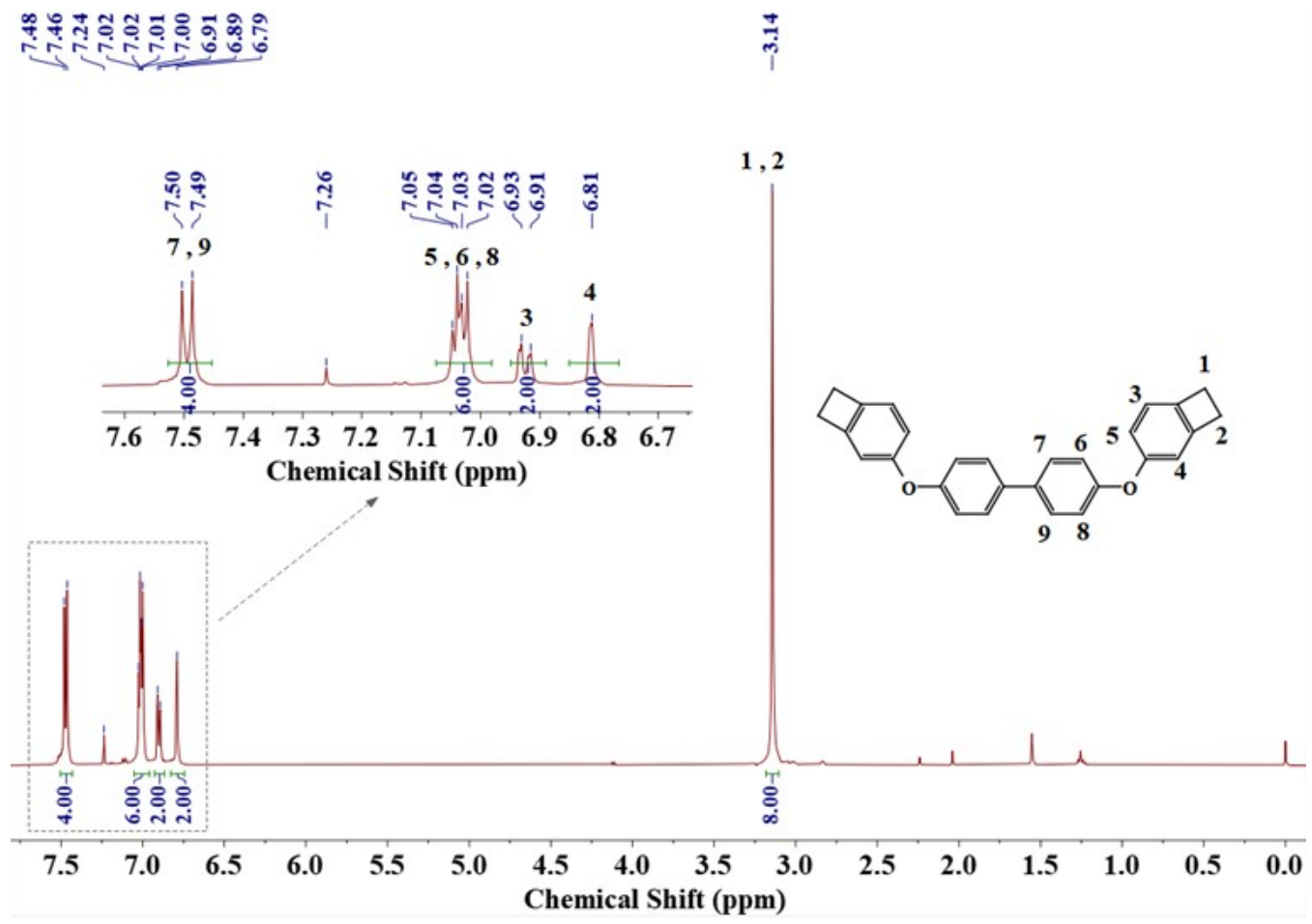

Fig. S6. ^{1}H NMR spectrum of the monomers **BCB-B** (500 MHz, $CDCl_3$).

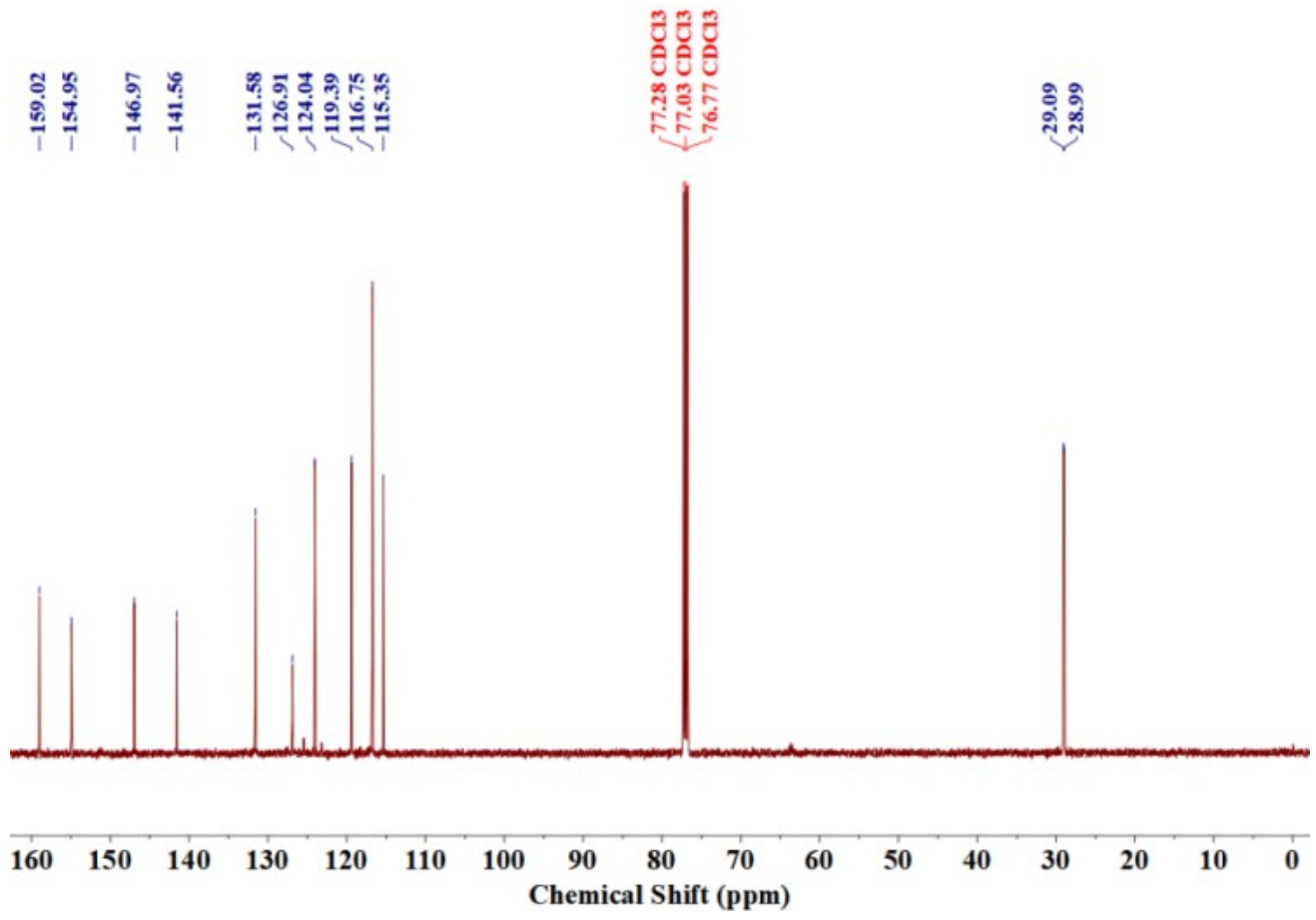

Fig. S7. ^{13}C NMR spectrum of the monomers **BCB-AF** (126 MHz, $CDCl_3$).

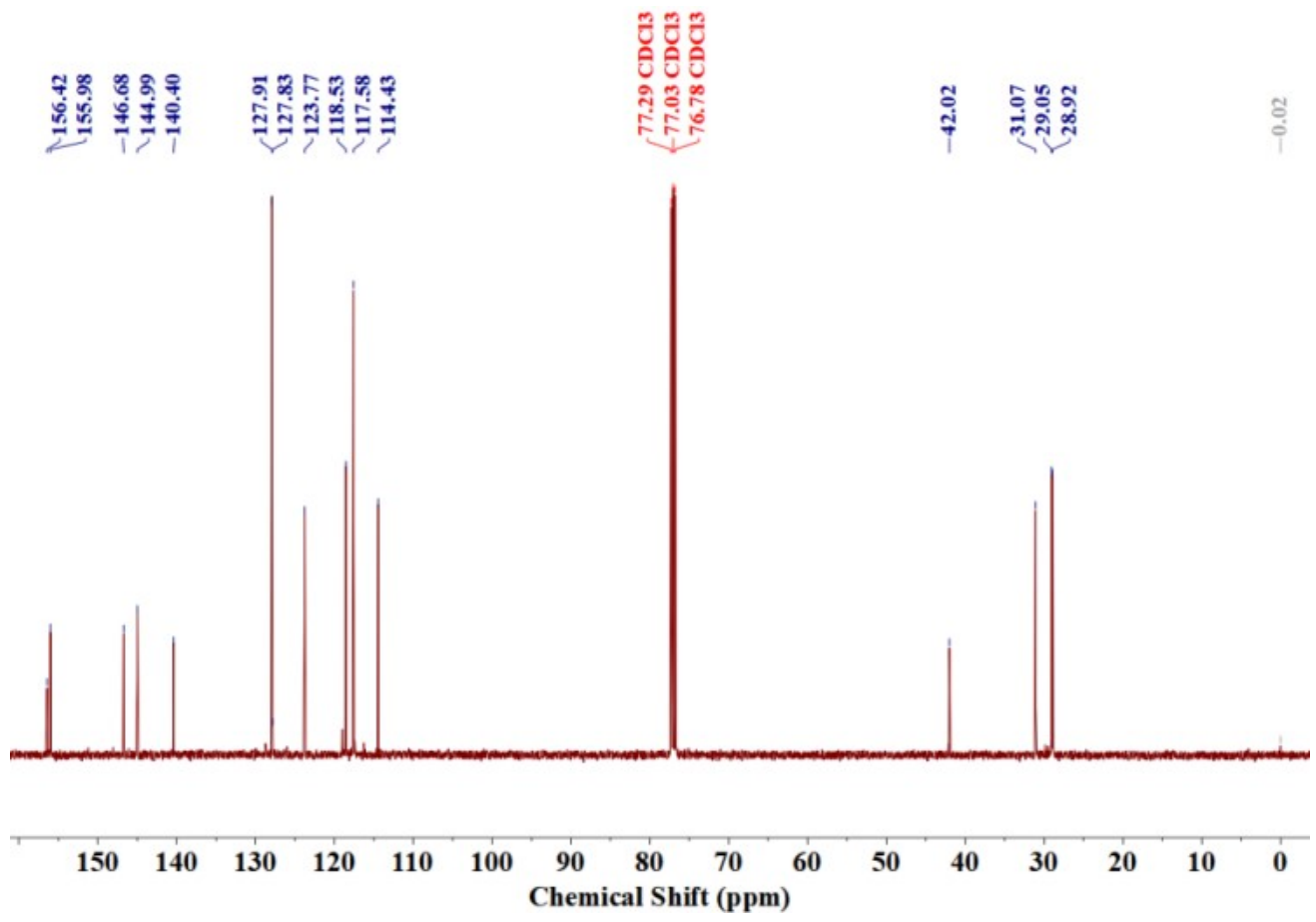

Fig. S8. ^{13}C NMR spectrum of the monomers **BCB-A** (126 MHz, $CDCl_3$).

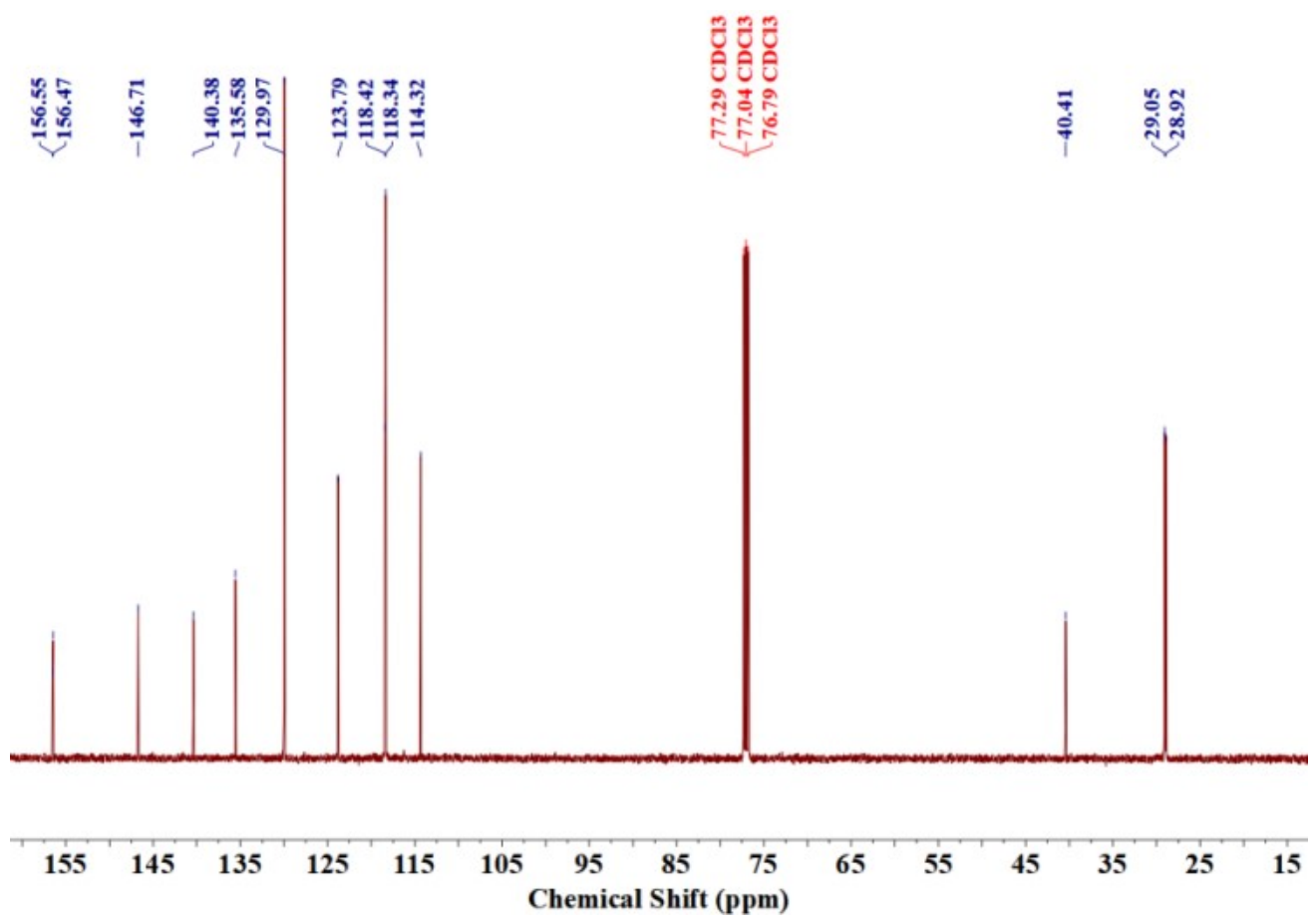

Fig. S9. ^{13}C NMR spectrum of the monomers **BCB-F** (126 MHz, $CDCl_3$).

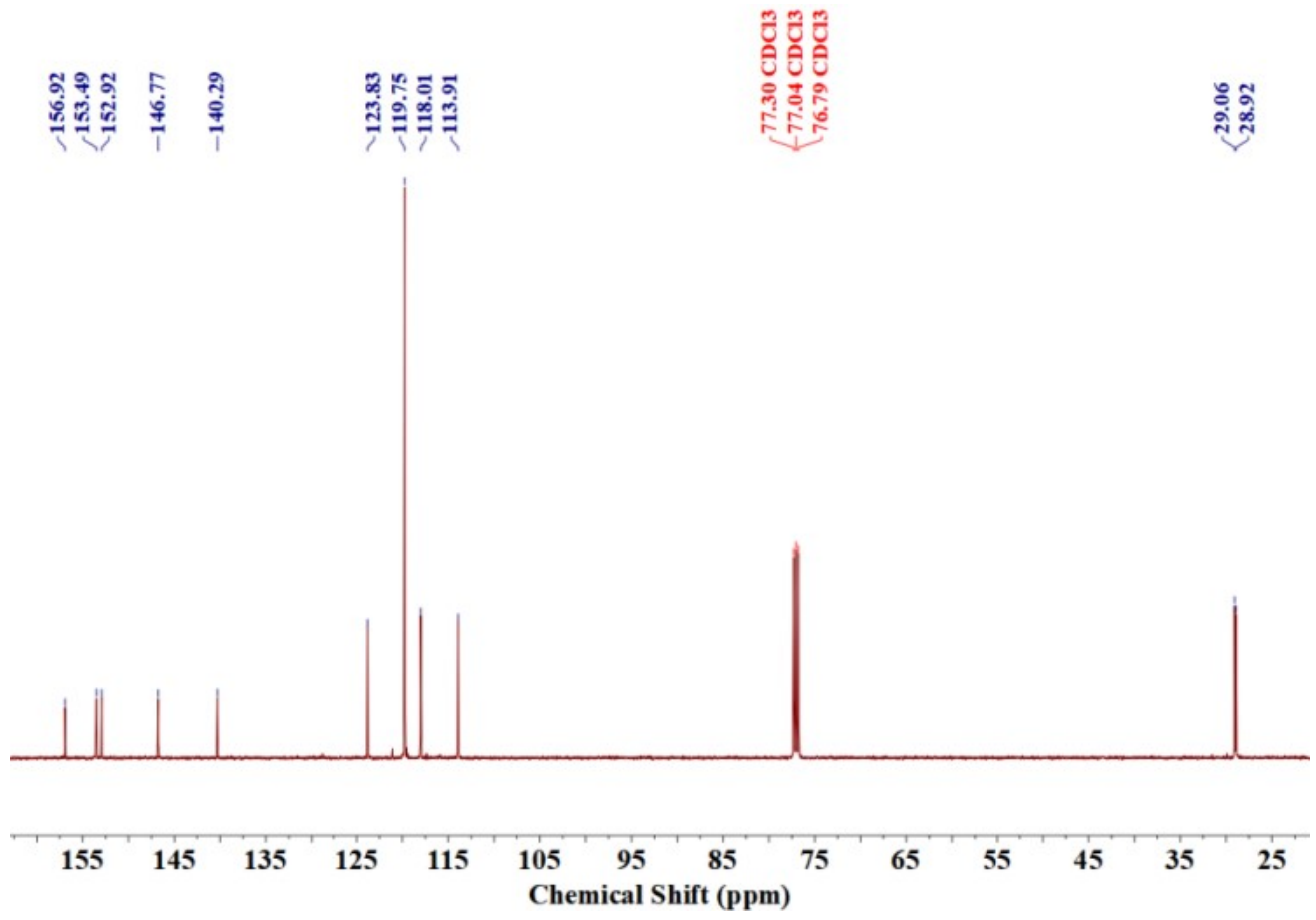

Fig. S10. ^{13}C NMR spectrum of the monomers **BCB-O** (126 MHz, $CDCl_3$).

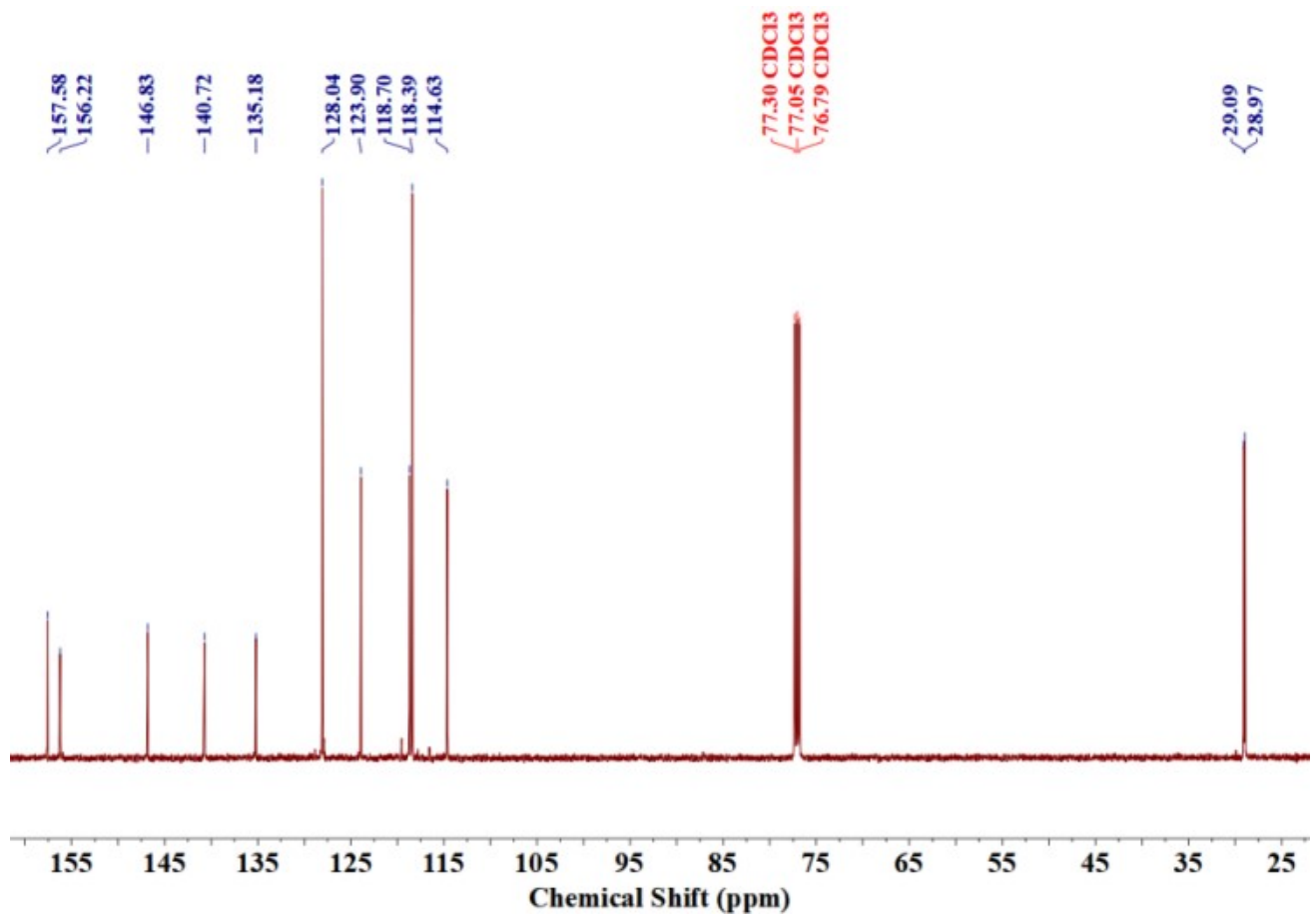

Fig. S11. ^{13}C NMR spectrum of the monomers **BCB-B** (126 MHz, $CDCl_3$).

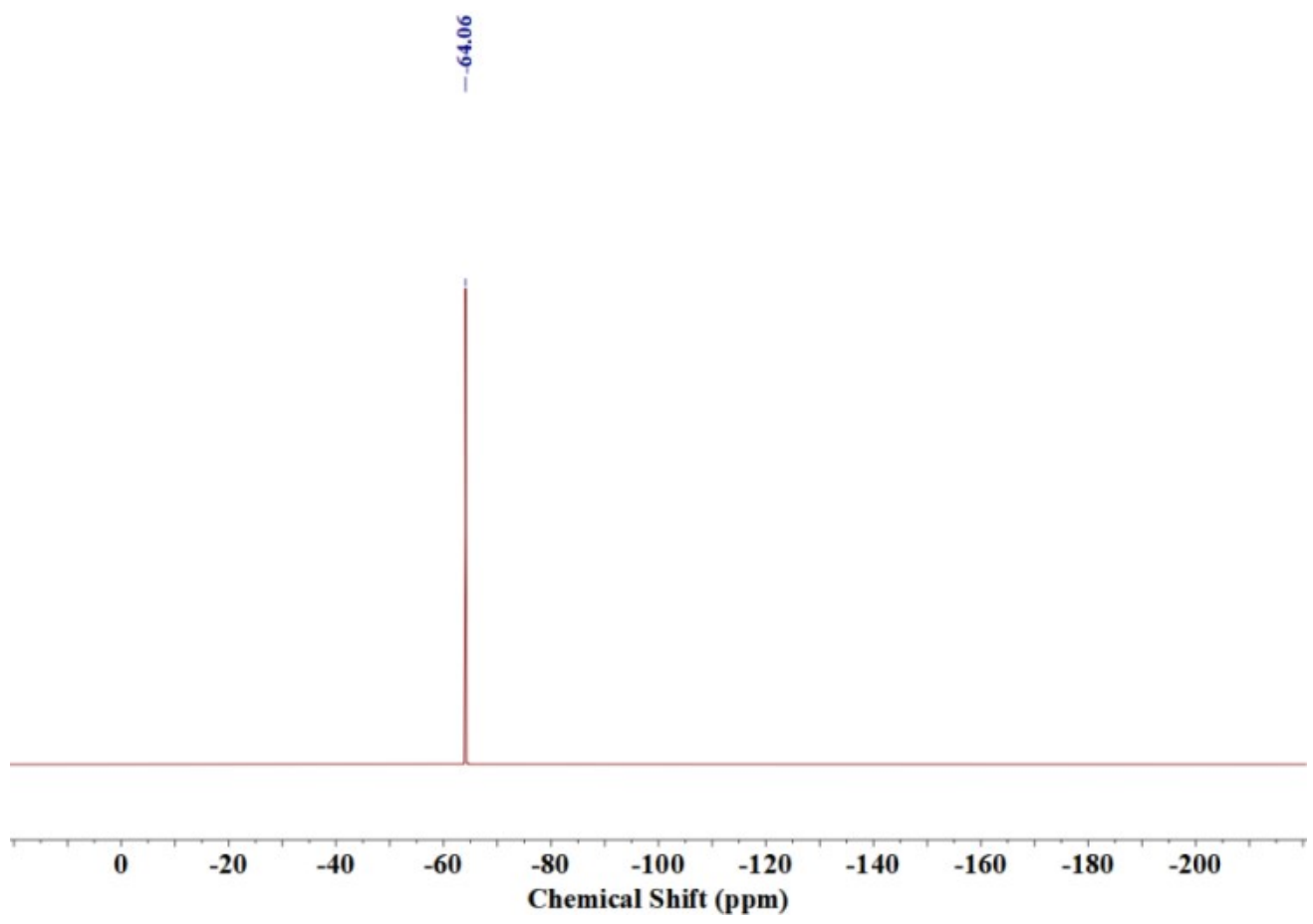

Fig. S12. ^{19}F NMR spectrum of the monomers **BCB-AF** (470 MHz, $CDCl_3$).

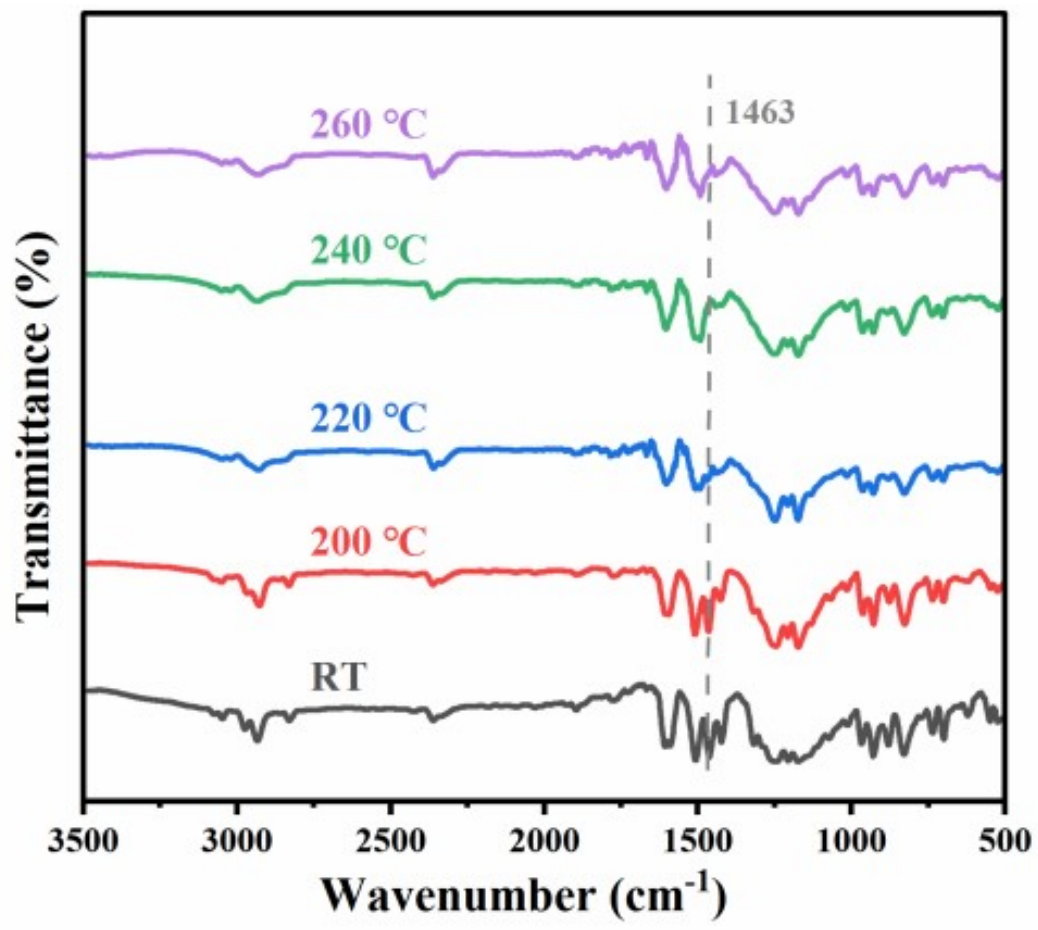

Fig. S13. FT-IR spectra of samples of **BCB-AF** precured at different temperatures for 1 h.

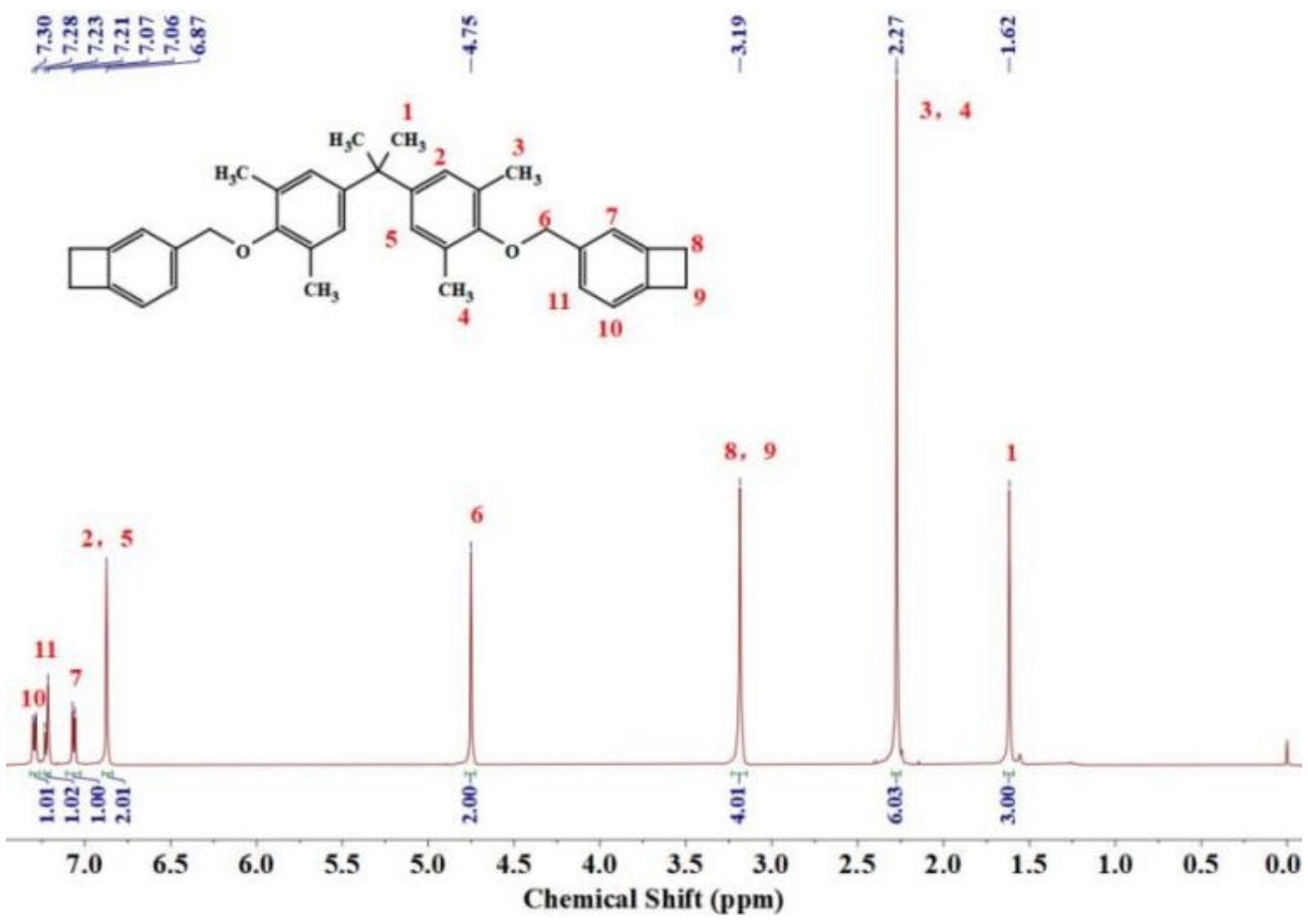

Fig. S14. ^{1}H NMR spectrum of the monomers **C-BPF** (500 MHz, $CDCl_3$).

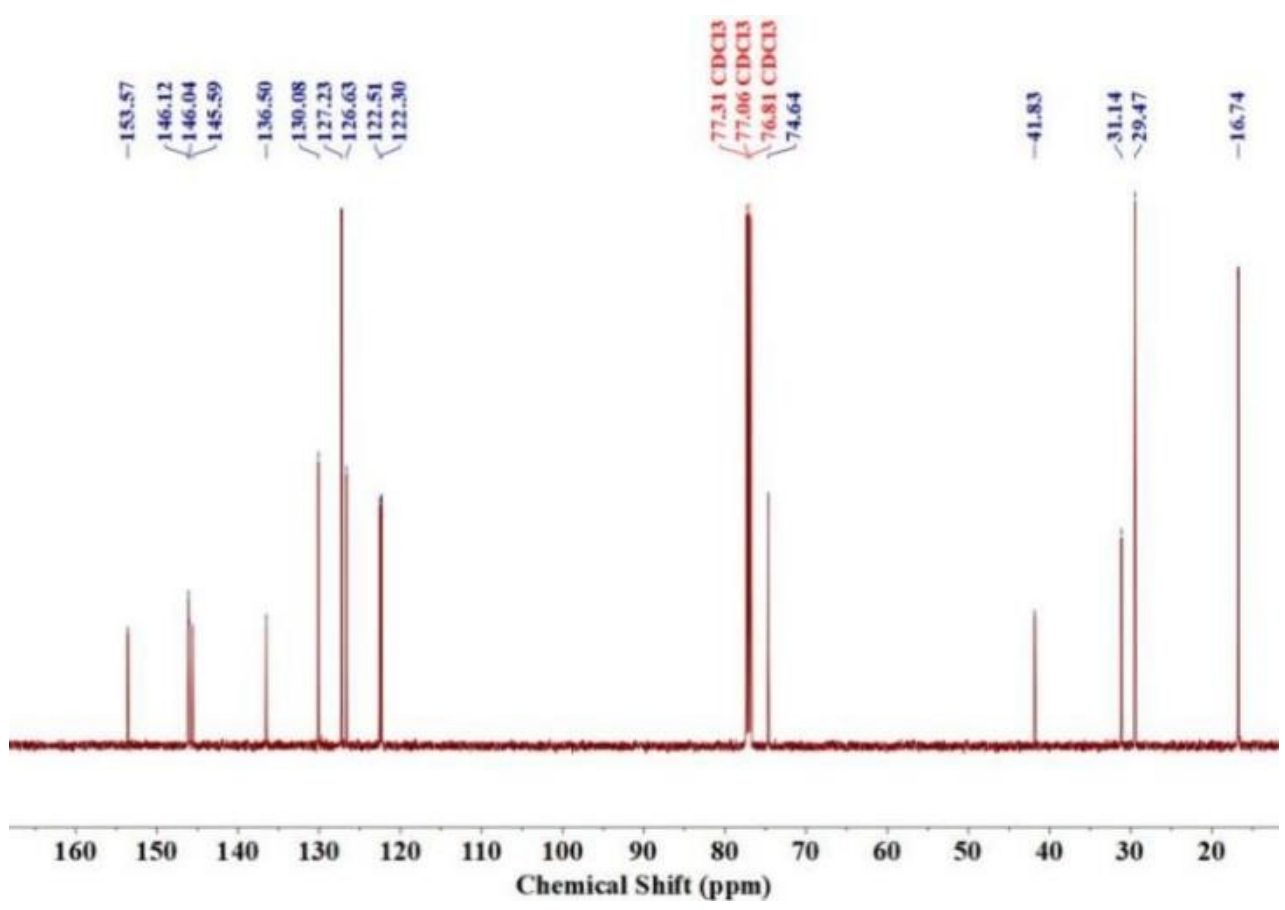

Fig. S15. ^{13}C NMR spectrum of the monomers **C-BPF** (126 MHz, $CDCl_3$).

References

1. Y. Zhai, et al., Copper-catalyzed diaryl ether formation from (hetero) aryl halides at low catalytic loadings, The Journal of Organic Chemistry, 2017, 82, 4964-4969.
2. L. Zhao, et al., Low dielectric polymers at high frequency with bulky adamantane groups as the linker, Polymer Chemistry, 2024, 15, 3063-3070.
3. S. Wu, et al., Preparation and performance of low-dielectric benzocyclobutene resins containing dicyclopentadiene, Journal of Vinyl and Additive Technology, 2022, 28, 631-639.
